# Seq2Pocket: augmenting protein language models for spatially consistent binding site prediction

**DOI:** 10.1093/bioinformatics/btag778

**Published:** 2026-10-16

**Authors:** Vít Škrhák, Lukáš Polák, Marian Novotný, David Hoksza

**Affiliations:** Department of Software Engineering, Faculty of Mathematics and Physics, Charles University, Prague, 118 00, Czech Republic; Department of Software Engineering, Faculty of Mathematics and Physics, Charles University, Prague, 118 00, Czech Republic; Department of Cell Biology, Faculty of Science, Charles University, Prague, 128 43, Czech Republic; Department of Software Engineering, Faculty of Mathematics and Physics, Charles University, Prague, 118 00, Czech Republic

## Abstract

**Motivation:**

Protein–ligand binding site prediction (LBS) is important for many areas of structural biology and molecular modeling, where, as in other tasks, protein language models (pLMs) have shown great promise. In their application to LBS, the pLM classifies each amino acid as binding or not, but translating these predictions into three-dimensional binding pockets remains challenging; in particular, residue-centric predictions tend to produce spatially fragmented pockets.

**Results:**

We present Seq2Pocket, a methodology for pocket-level LBS prediction that combines pLM finetuning, data enhancement, and structure-aware post-processing. First, we introduce sc-PDB_enhanced_, an extended training dataset that augments an existing sc-PDB dataset with additional small-molecule and ion-binding sites, improving coverage of non-obvious and small pockets. Second, we employ an embedding-supported smoothing classifier to refine residue-level predictions. Third, we define the Pocket Fragmentation Index and use it to select a clustering approach that preserves a consistent mapping between predictions and ground-truth pockets. We evaluate Seq2Pocket on two tasks: general binding site prediction using the LIGYSIS benchmark and cryptic binding site prediction using CryptoBench benchmark. Across both benchmarks, Seq2Pocket achieves state-of-the-art DCC_top-N_ and DCC_top-(N + 2)_ performance. In particular, for general binding site prediction on the LIGYSIS benchmark, it improves distance-center-to-center recall by up to 13%, outperforming existing predictors. This improvement originates from training data quality rather than model architecture, as demonstrated by an ablation study. We believe that these findings contribute to more reliable evaluation practices in ligand binding site prediction, highlight the importance of training data curation, and provide pocket-level prediction tools that are better suited for downstream applications such as drug discovery.

**Availability:**

The Seq2Pocket web interface is available at https://seq2pocket.projekty.ms.mff.cuni.cz, running the ESM-2 650M variant. The sc-PDB_enhanced_ is published using Zenodo platform at https://zenodo.org/records/18271517. The models are published at HuggingFace: https://huggingface.co/skrhakv/seq2pocket.

## Graphical Abstract

**Graphical Abstract:**
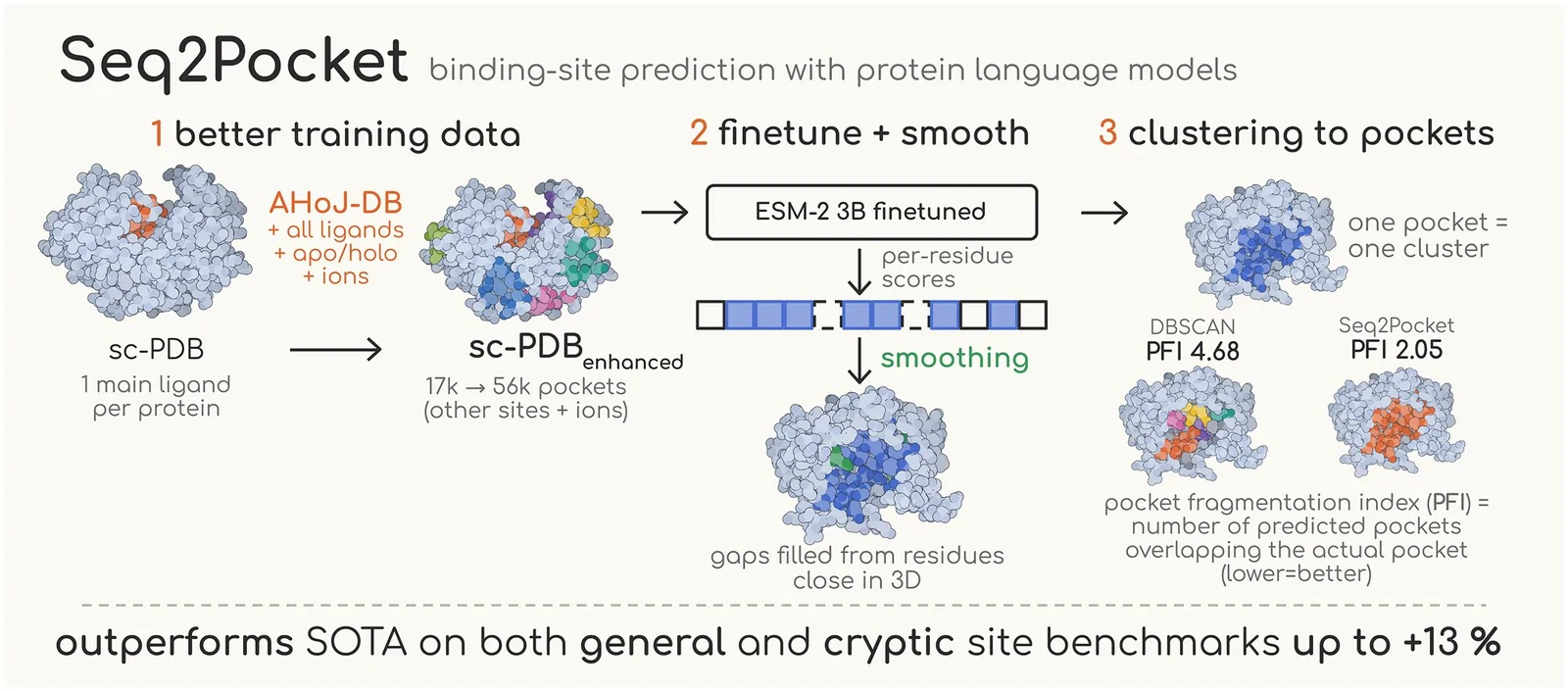

## 1 Introduction

Proteins drive most biological processes, so understanding how they work, and how to control their activity, lies at the center of fields like drug discovery, biotechnology, and agriculture. Direct experiments, whether *in vitro* or *in vivo*, take time and resources, which limits how far they can be pushed. That constraint has motivated many researchers toward *in silico* approaches, which offer a faster and more affordable way to study proteins (Jiménez-Luna *et al.* 2021).

One of the main ways to control the function of a protein is through the binding of small molecules, often called ligands. This strategy is highly successful in drug discovery, as nearly 90% of current drugs are small-molecule ligands (Southey and Brunavs 2023). The challenge is finding where these ligands can bind those sites. Over the years, many computational approaches have been developed to find and examine these ligand binding sites (LBS), including Molecular Dynamics (MD) simulations (Abraham *et al.* 2015, De Vivo *et al.* 2016), energy-based methods (Laurie and Jackson 2005, Halgren 2009, Kozakov *et al.* 2015), docking (Ferreira *et al.* 2015), and machine learning (Zhao *et al.* 2020, Utgés and Barton 2024, Gašparíková *et al.* 2025).

Recent advances in deep learning have reshaped structural biology in two main directions. One is the emergence of structure prediction systems such as AlphaFold (Jumper *et al.* 2021). The other is the development of protein language models (pLM) (Heinzinger *et al.* 2019, Elnaggar *et al.* 2022, Lin *et al.* 2023, Heinzinger *et al.* 2024). When combined with techniques such as transfer learning and finetuning, pLMs reach or exceed the performance of existing methods on several downstream tasks, including variant effect prediction (Pugh *et al.* 2025), protein function prediction (Wang *et al.* 2025a), and enzyme activity prediction (Wang *et al.* 2025b).

Naturally, ligand binding site (LBS) prediction has also been influenced by the recent advances in deep learning (Li *et al.* 2019, Zhao *et al.* 2020). Since the introduction of protein language models (pLMs), a growing number of deep learning–based methods have incorporated pLM representations for LBS prediction (Škrhák *et al.* 2023, Carbery *et al.* 2024, Sestak *et al.* 2024, Gamouh *et al.* 2025, Škrhák *et al.* 2025). This shift toward pLM-based approaches aligns closely with the structure of the problem: pLMs have been shown to encode local motifs (Zhang *et al.* 2024), and binding pockets can be, to a large extent, interpreted as local motifs on the protein sequence. In typical pLM-based binding site prediction pipelines (i.e. *transfer learning* through embeddings, or *finetuning*), the model produces a per-residue score that reflects the probability of that residue belonging to a binding site. Despite the competitive performance reported for many of these approaches, a recent independent evaluation suggests that traditional methods continue to perform strongly in LBS prediction, in some cases matching or outperforming newer deep learning–based techniques (Utgés and Barton 2024).

When comparing the residue-level and pocket-level performance, we can find a conflict between these two metrics: while residue-level metrics might look promising, especially in cases where a large binding site provides many true positive residues, the pocket-level metrics could be disappointing. For example, if a protein has three distinct binding pockets and a predictor only finds the largest one, its pocket-level recall is only 33%, even if its residue-level scores are high.

Therefore, while residue-level performance is a prerequisite of a good predictor, it is insufficient for binding site discovery evaluation. On that account, pocket-level metrics such as distance-center-to-center (**DCC**) have become the evaluation standard (Krivák and Hoksza 2018, Utgés and Barton 2024).

In this study, we identify three challenges related to LBS evaluation and the use of pLMs:

1. *Spatial inconsistency*: A truly robust LBS predictor should identify continuous binding regions rather than scattered residues. We observe a tendency in pLMs to produce what we define as **incomplete pockets**: residue-wise predictions that often achieve high statistical scores but fail to form continuous binding regions (see examples in Figs. 1, 2 and Fig. S2a, S3a in the Supplementary Material).
2. *Bias towards large binding sites:* Current literature suggests that most predictors excel at identifying large orthosteric sites while struggling with smaller, more challenging targets (Utgés and Barton 2024). We argue that this performance issue is largely driven by limitations in existing training sets. We further show that augmenting the training data with more diverse binding conformations significantly improves the detection of these smaller sites.
3. *Metrics inflation via clustering fragmentation*: Mapping residue-level predictions to three-dimensional pockets requires a clustering step. We demonstrate that the choice of clustering significantly impacts pocket-level results; while one approach may degrade performance, another can paradoxically make a model appear superior by inflating pocket-level metrics like DCC. This occurs when the algorithm partitions a single binding region into numerous small clusters: a phenomenon we refer to as **clustering fragmentation**. While such fragmentation may improve numerical scores (DCC), it does not correspond to better identification of functional binding sites and reduces the practical usefulness of the predictions.

**Figure 1:**
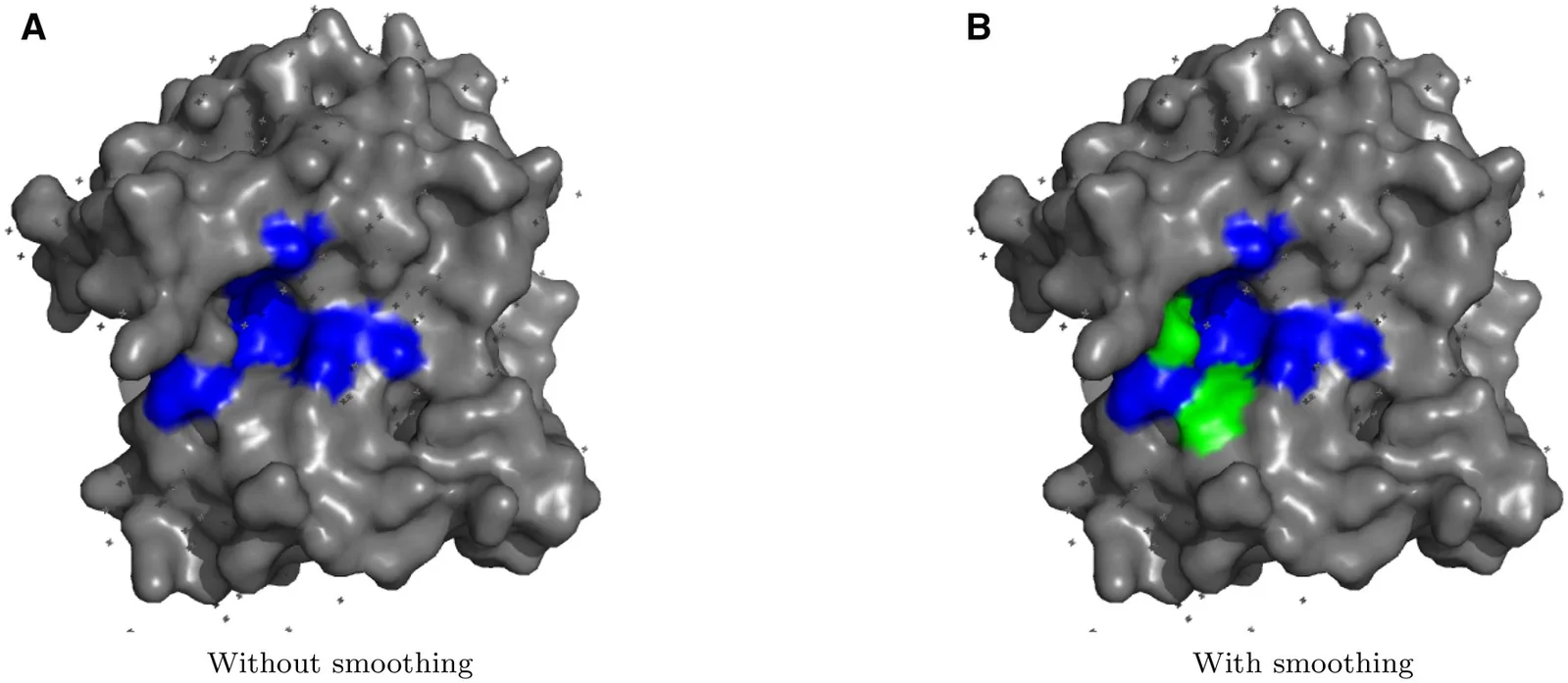
Example of residues added by smoothing classifier (GREEN: Pro8, Phe56, Ile58, Val166) of a predicted pocket (BLUE). This is a 6isuA structure.

**Figure 2:**
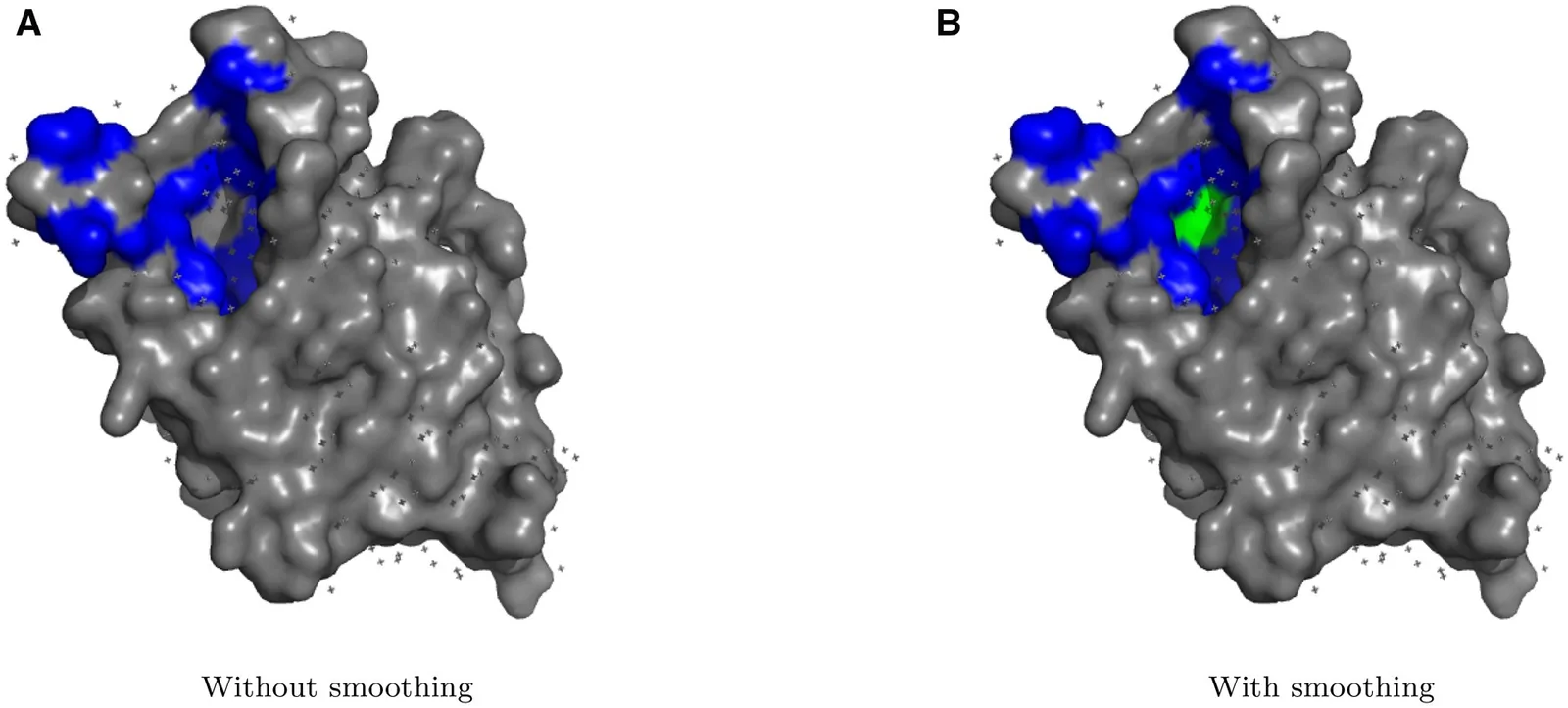
Example of residues added by smoothing classifier (GREEN: Thr1042, Thr1067) of a predicted pocket (BLUE). The cavity was not fully predicted by the finetuned pLM. The smoothing classifier fills the gap. This is a 5yqpA structure.

To address these challenges, we develop a prediction methodology named **Seq2Pocket** built upon a finetuned ESM-2 pLM, that serves as the methodology’s prediction core. This methodology contributes in three ways. First, we address the problem of **incomplete pockets** by introducing an embedding-supported smoothing classifier that uses latent representations of neighboring residues to refine residue-level predictions, ensuring structurally complete binding pockets (see Section 2.2). Second, to improve performance toward non-obvious targets, we construct **sc-PDB_enhanced_** dataset that incorporates *small-molecule-binding* and *ion-binding* sites in order to reduce their omission during inference (see Section 2.4). Third, we define the **Pocket Fragmentation Index** (see Section 2.5), and conduct a comparison of different clustering strategies.

We evaluate our approach on two challenges: the prediction of general binding sites (GBS) and the prediction of cryptic binding sites (CBS). In both challenges, Seq2Pocket achieves the best DCC_top-N_ and DCC_top-(N + 2)_ among the compared methods.

## 2 Methods and materials

Our methodology consists of a multi-stage pipeline. At its core is a finetuned ESM-2 (3B) model that serves as the residue-level prediction core. For GBS prediction, we train two variants of this core to quantify the impact of training data quality: one on the standard sc-PDB dataset (Desaphy *et al.* 2015) and another on our augmented sc-PDB_enhanced_ (2.4 Datasets). For CBS prediction, we utilized an existing model finetuned on the CryptoBench training set (Škrhák *et al.* 2025). Predicted residue scores are then refined using the embedding-supported smoothing classifier. Finally, spatial clustering is applied to group residues into binding pockets.

### 2.1 pLM finetuning

The GBS prediction task required finetuning of the ESM2-3B protein language model on the scPDB dataset. We first adapted the model architecture by removing the masked language modeling head and replacing it with a binary classification head operating at the residue level. Training was performed using binary cross-entropy loss with the AdamW optimizer and L2 regularization. A dropout rate of 0.3 (Srivastava *et al.* 2014) was applied, and the learning rate was set to 1e − 4. Due to GPU memory constraints, the batch size was fixed to one. The model was finetuned for a total of 3 epochs, with class weighting employed to stabilize the imbalance between binding and non-binding residues (9.40% of positives in the LIGYSIS dataset). The finetuning was performed in two stages. In the first stage, the weights of the entire pLM backbone were frozen, allowing only the newly initialized binary classification head to be trained. In the second stage, the entire model (backbone and head) was unfrozen and trained jointly. The threshold for assigning the positive class was set to 0.7. All parameters were set based on a validation run using one of the CryptoBench train splits as a validation set.

The whole architecture is depicted in Fig. S5 in the Supplementary. Further details on hyperparameter tuning can be found in the Supplementary Section 3. We also finetuned the 650M variant of ESM2 for availability on hardware with restricted resources.

For the CBS task, a finetuned ESM2-3B model from a recent study (Škrhák *et al.* 2025) was employed without any adjustments.

### 2.2 Pocket smoothing

We introduce a binary classifier designed to decide whether a candidate residue should be incorporated into a predicted binding pocket. The purpose is to recover residues that are missed in the initial residue-level prediction.

The classifier is implemented as a three-layer feed-forward neural network. It consists of two hidden layers with 2048 neurons each, followed by a single-neuron output layer with a sigmoid activation for binary classification. Training is carried out for 19 epochs using a batch size of 512 and a dropout rate of 0.5. We optimize the model using binary cross-entropy loss with the AdamW optimizer and L2 regularization. Class weighting is applied to counter the imbalance between positive and negative examples. Hyperparameters are selected based on validation experiments on one of the training subsets, optimizing the area under the precision–recall curve (AUPRC).

Each input to the classifier is formed by concatenating two embeddings: an embedding of the candidate residue and a pocket context embedding. Both are taken from the final hidden layer of the ESM-2 (3B) protein language model.

The pocket context embedding is intended to represent the local environment of a predicted binding site. For a given candidate residue, we identify all neighboring residues within a 15 Å radius that were initially predicted as binding residues by the pLM. The embeddings of these neighboring residues are then aggregated using mean pooling to produce the pocket context embedding.

The classifier is trained using annotated binding sites from the CryptoBench dataset. Training samples are constructed as follows:

- *Positive Examples:* residues that belong to a known binding site.
- *Negative Examples:* non-binding residues located within 10 Å of a binding site.

During inference, a negatively predicted residue is considered for smoothing if it lies within 15 Å from any positively predicted residue.

The geometric thresholds were determined using one of the CryptoBench training splits as a validation set. An overview of the pocket smoothing architecture is provided in Fig. S6 in the Supplementary Material, together with further details on hyperparameter tuning in Supplementary Section 3.

### 2.3 Clustering

After residue-level prediction and smoothing, predicted binding residues are grouped into discrete three-dimensional regions using a surface-based clustering procedure. For each atom belonging to a predicted binding residue, we generate solvent-accessible surface (SAS) points using the Shrake–Rupley algorithm (Shrake and Rupley 1973), with a probe radius of 1.6 Å and a point density of 50. We employ a modified implementation from the Biopython library (Cock *et al.* 2009). The resulting SAS point cloud is then clustered into candidate binding pockets using the Mean Shift algorithm (Cheng 1995) with a bandwidth of 9 Å (see Supplementary Section 3: hyperparameter tuning). An example of the resulting SAS point distribution for a correctly predicted binding site on human C-terminal Src kinase [PDB ID: 1BYG, chain A (Lamers *et al.* 1999)], where the Staurosporine inhibitor binds, is shown in Fig. S1 in the Supplementary Material.

Each surface-based cluster was then mapped back to residues using a voting scheme: each solvent-accessible surface (SAS) point inherits the cluster assignment produced by the Mean Shift algorithm, and each residue contributes multiple SAS points. If a predicted residue has no SAS points, the C-alpha coordinates are used for clustering instead. A residue is assigned to the cluster for which the majority of its SAS points vote. Once residues are assigned to clusters, each resulting pocket is scored. The score is the sum of squares of the predicted binding probabilities of its residues, as provided by the finetuned pLM. These pocket scores are subsequently used to rank the predicted binding sites.

### 2.4 Datasets

This work utilizes four datasets (Table 1):

**Table 1:** Datasets statistics.

Dataset	Purpose	# Of structures	# Of residues	# Of binding residues	% Of binding residues (%)	# Of pockets
LIGYSIS	GBS evaluation	2775	832 071	78 205	9.40	6882
sc-PDB	GBS training	17 077	5 558 460	536 011	9.64	17 077
sc-PDB_enhanced_	GBS training	17 077	5 558 460	824 046	14.83	56 968
sc-PDB_enhanced-f_	GBS training	13 140	4 375 626	607 985	13.89	38 578
CryptoBench test subset	CBS evaluation	184	55 572	3158	5.68	213
CryptoBench train subset	CBS training	762	233 435	13 342	5.72	872

LIGYSIS and CryptoBench test sets were used for evaluation, sc-PDB and CryptoBench train sets were used for training. Although the sc-PDB and sc-PDB_enhanced_ datasets contain the same structures, the sc-PDB_enhanced_ contains additional pockets found either in that structure or in a different conformation of the same protein. The sc-PDB_enhanced-f_ row marks the subset after the removal of identical UniProt IDs against LIGYSIS, which was later used for the Seq2Pocket (GBS) training.

- **sc-PDB** for training the GBS predictor,
- **LIGYSIS** for evaluating the GBS predictor,
- **COACH420** for evaluating the GBS predictor,
- **CryptoBench** for evaluating the CBS predictor.

#### 2.4.1 LIGYSIS dataset

The LIGYSIS dataset is the most recent dataset of ligand binding sites. It contains non-redundant protein–ligand interfaces. To remove redundancy, as well as take advantage of all available data, the annotations were mapped to a single representative biological unit using UniProt mapping. The LIGYSIS also includes ion binding sites; in fact, about 20% of the pockets are binding ions. We used its subset of 2775 human protein chains, which was utilized in a recent study to evaluate the performance of 11 ligand-binding site predictors (Utgés 2024). This allowed us a direct comparison with these evaluated methods.

#### 2.4.2 sc-PDB dataset

The sc-PDB dataset is one of the most widely used datasets for training binding site prediction methods (Kandel *et al.* 2021, 2024, Aggarwal *et al.* 2022, Sestak *et al.* 2024, Smith *et al.* 2024). We used sc-PDB v2017, the most recent version, which contains 17 077 structures (The whole dataset contains 17 594 structures, but due to annotation mismatches, we were able to retrieve only 17 077 structures.). sc-PDB has been reported to have 9.7% overlap with the LIGYSIS dataset (Utgés and Barton 2024). Unfortunately, the original sc-PDB dataset considers only the most relevant ligand for each entry. This is not biologically accurate because a single protein can contain more than one binding site. Furthermore, sc-PDB does not consider ion binding sites.

We therefore extended sc-PDB using AHoJ-DB (version *v2c*) (Feidakis *et al.* 2022, 2024), a PDB-wide database of ligand binding sites and their apo/holo states (see Supplementary Section 2 for a brief overview of AHoJ-DB relevant to this work). For every sc-PDB structure and chain, we identified all AHoJ-DB-annotated ligand binding sites reported for that chain across all ligands and across both its apo and holo conformations and merged their annotated residues, together with the original sc-PDB annotation, into a single per-chain set of binding residues. Each residue in the chain is labeled as binding if it belongs to this merged set and non-binding otherwise. These per-residue labels are used for residue-level training. We refer to the resulting dataset as sc-PDB_enhanced_. This expansion increased the total number of pockets from 17 077 to 56 968 (Table 1). The full construction pipeline, including the AHoJ-DB structure-selection criteria and manual corrections, is described in Supplementary Section 2.

We note that the reported 9.7% pocket overlap between sc-PDB and the LIGYSIS test set is problematic. This overlap also affects sc-PDB_enhanced_, since its additional pockets are derived from the same underlying protein structures. Therefore, in any subsequent training, we removed the overlapping UniProt sequences from the training data in both sc-PDB and sc-PDB_enhanced_, based on chain-to-UniProt mapping obtained from the PDBe API. As a result, the number of protein structures used for training was reduced from 17 077 to 13 140 reported as sc-PDB_enhanced-f_ in Table 1. Correspondingly, the total number of annotated pockets was reduced from 17 077 to 13 140 in sc-PDB and from 56 968 to 38 578 in sc-PDB_enhanced_. All training experiments reported in this work use these filtered datasets.

#### 2.4.3 CryptoBench dataset

The CryptoBench dataset (Škrhák *et al.* 2024) is built on top of AHoJ-DB (Feidakis *et al.* 2022, 2024) via a multi-stage processing pipeline, including structural quality check filtering (TM-score ≥ 0.5), resolution filtering (≤ 2.5 Å) and ensuring geometric compactness of pockets by controlling the radius of gyration (max. 20% change). Most importantly, it applies a crypticity criterion based on pocket RMSD. Specifically, the pocket is marked as cryptic, if there exists a conformation where the RMSD of the pocket atoms between ligand-bound state (holo) and the ligand-free state (apo) exceeds 2 Å. To remove redundancy, the resulting structures are filtered down to 40% sequence identity (Steinegger and Söding 2017). The dataset provides pre-defined training and test splits: to ensure no information leakage occurs between them, we applied a secondary filter to the remaining sequences once again, this time to 10% sequence identity, meaning that no two sequences from different subsets share more than 10% sequence identity. It should be noted that CryptoBench does not include ion binding sites. In this work, we used a subset of CryptoBench that excludes binding sites spanning multiple chains.

#### 2.4.4 COACH420 dataset

COACH420 (Krivák and Hoksza 2018) is a benchmark for ligand binding site prediction, derived from the original COACH test set (Roy *et al.* 2012, Yang *et al.* 2013). Prior to the introduction of LIGYSIS (Utgés and Barton 2024), it served as one of the most widely used test sets for evaluating binding site predictors. However, COACH420 lacks redundancy controls used in LIGYSIS, and is comparatively small and less diverse than LIGYSIS. We include it here for completeness and comparability with prior literature; corresponding results are reported in the Supplementary Material.

### 2.5 Evaluation framework

Our evaluation uses two benchmarks: the LIGYSIS dataset (Utgés and Barton 2024) for GBS and CryptoBench (Škrhák *et al.* 2024) test set for CBS. Each example in these datasets can be derived from multiple structural sources. For LIGYSIS, binding site annotations are often aggregated from several PDB structures; we follow the dataset’s convention by utilizing the predefined representative structure and chain ID during the evaluation (see Supplementary Section 4: “Selection of methods for comparison” for further details on how methods for comparison were selected). In the case of CryptoBench, binding sites are provided in both apo (ligand-free) and holo (ligand-bound) forms (Brylinski and Skolnick 2008). We perform our evaluation exclusively on the apo structures, as this represents the most relevant scenario for the discovery of the binding site, where the bound conformation is unknown and the predictor must identify the site on an unbound protein surface.

We report standard classification metrics at the residue-level: Matthews Correlation Coefficient (MCC), Area Under the ROC Curve (AUC), Area Under the Precision-Recall Curve (AUPRC), F1-score, and Accuracy (ACC). As the datasets are heavily imbalanced (only 5% are positive examples in some datasets), MCC, AUPRC, and AUC serve as more robust performance indicators. While ACC and F1-score are reported for completeness, they are less optimal for highly imbalanced datasets (Chicco and Jurman 2020).

The main focus of this study is on pocket-level metrics, specifically **DCC-based (distance-center-to-center) recall** and **Relative Residual Overlap** (**RRO**). DCC measures the distance between the geometric centers of the predicted binding site and the ground-truth binding site. A prediction is a ’hit’ if the distance between the predicted and ground-truth pocket centers is **below 12 Å** (Utgés and Barton 2024).

To test how well the model ranks its predictions, we calculate recall using different selection limits. For a protein with *N* experimentally known sites **DCC_top-N_** considers only the *N* highest-scoring predicted pockets. However, protein datasets are often sparsely annotated; a model might correctly identify a valid but unannotated site as its top prediction, which would push a known site out of the list of top-*N* results. To account for this, we report **DCC_top-(N + K)_**, where we set K = 2 to provide a budget of two additional predictions. This metric assesses whether the N ground-truth pockets are successfully identified within the model’s top N + K candidates. Finally, **DCC_MAX_** considers all predicted pockets regardless of N; while this provides an upper bound for recall, it should be interpreted alongside precision to avoid favoring models that over-predict or over-fragment their predictions.

Next, RRO was computed for each observed (actual) pocket from the evaluation dataset as


(1) $$\mathrm{RRO}=\frac{\left|P\cap A\right|}{\left|A\right|}$$

where *A* is the set of residues in the actual pocket and *P* is the set of residues in the predicted pocket matched to it by minimum center-to-center distance. RRO was recorded only for pockets whose matched prediction had a DCC below 12 Å (i.e. correctly predicted pockets); undetected pockets were excluded.

To evaluate the clustering method, we suggest a measure called Pocket Fragmentation Index (PFI), which measures the average number of predicted clusters assigned to each ground-truth pocket:


(2) $$\mathrm{PFI}=\frac{1}{N}\sum_{i=1}^{N}|P_{i}|$$

where *N* is the total number of ground-truth pockets and $\left|P_{i}\right|$ is the number of predicted clusters that overlap the i-th actual pocket. An ideal clustering strategy achieves a one-to-one mapping, resulting in a PFI of 1.0. By monitoring this index, we can identify **excessive clustering fragmentation**, where a single biological binding site is incorrectly partitioned into multiple predicted regions. A high PFI indicates that, while the residues may be correctly predicted, the structural representation is partitioned into too many segments, making later downstream applications more difficult. Partitioning the binding site into more fragments can also artificially help inflating the DCC-based metrics.

Although cryptic binding sites show structural flexibility, they typically exhibit localized conformational changes, such as loop rearrangements or secondary structure displacements, rather than a total pocket reorganization (Beglov *et al.* 2018, Vajda *et al.* 2018). Therefore, the same structural metrics (DCC-based recall, coverage, and PFI) remain applicable for the CBS task.

The evaluation framework for the GBS task is identical to that of Utgés and Barton (2024), which allows direct comparison between our results and other state-of-the-art methods measured on the LIGYSIS dataset. We report only the best-performing methods for comparison in both residue-level and pocket-level metrics. Similarly, for the CBS task, the performance at the residue-level of the transfer learning baseline is sourced from Škrhák *et al.* (2025), as the study uses the same evaluation dataset.

## 3 Results

We present Seq2Pocket, a pocket-level binding site prediction method built on a finetuned pLM core, combined with training-data assembly (Only for the GBS task.) and structure-aware post-processing. Prior work has shown that geometry-based methods such as P2Rank and fpocket can outperform many deep learning approaches (Utgés and Barton 2024). We therefore compare Seq2Pocket against existing predictors on both tasks, using the LIGYSIS evaluation framework (Utgés and Barton 2024) for GBS, which covers the most comprehensive set of predictors evaluated to date.

We conducted an ablation study in the GBS task with enhancing the training dataset vs. without enhancing the training dataset to see the effect of the improved training data.

We also conducted an ablation study for both the GBS and CBS models by evaluating each in two configurations: without pocket smoothing and with the proposed pocket smoothing classifier.

### 3.1 Residue-level evaluation

Table 2 summarizes the residue-level performance in the LIGYSIS and CryptoBench evaluation sets. Although Accuracy (ACC) and F1-score are reported for completeness, the main focus of this evaluation is on MCC, AUC, and AUPRC, as these metrics are more robust to class imbalance.

**Table 2:** Residue-level performance comparison across GBS and CBS tasks.

Evaluation	Method	Train set	AUPRC	AUC	MCC	ACC (%)	F1
CryptoBench test set (CBS)	Seq2Pocket	CryptoBench train	**0.475**	**0.894**	0.460	92.4	0.932
	Seq2Pocket (without smoothing)	CryptoBench train	**0.475**	**0.894**	**0.473**	93.5	0.939
	CryptoBench baseline (Škrhák et al. 2025)	CryptoBench train	0.382	0.871	0.395	93.1	0.933
LIGYSIS (GBS)	Seq2Pocket	sc-PDB_enhanced_	**0.552**	**0.853**	0.438	87.2	0.884
	Seq2Pocket (without smoothing)	sc-PDB_enhanced_	**0.552**	**0.853**	**0.468**	89.4	0.899
	Seq2Pocket	sc-PDB	0.490	0.816	0.410	88.4	0.889
	PUResNet (Kandel et al. 2021, Utgés and Barton 2024)	sc-PDB	–	–	0.39	92.7	0.917
	P2Rank_CONS_ (Krivák and Hoksza 2015, 2018, Utgés and Barton 2024)	CHEN11	0.46	0.76	0.30	85.9	0.870
	fpocket (Utgés and Barton 2024)	–	–	–	0.12	52.4	0.616
	Ligsite (Hendlich et al. 1997, Utgés and Barton 2024)	–	0.38	0.70	0.21	74.7	0.795
	Only top-performing methods from (Utgés and Barton 2024) are shown, but Seq2Pocket (GBS) is implicitly compared against all methods in that study						

The table compares our proposed finetuning and smoothing approach against state-of-the-art baselines using residue-level metrics. Performance metrics for *Baseline transfer learning, PUResNet, and P2Rank_CONS_* are adopted from existing benchmarks (Utgés and Barton 2024, Škrhák *et al.* 2025). Note that for the GBS task, only the top-performing methods from the LIGYSIS study (Utgés and Barton 2024) are included for comparison. Primary emphasis is placed on MCC, AUC, and AUPRC; note that AUC and AUPRC are invariant to the smoothing process as it modifies binary labels without altering the underlying probability distributions. As *PUResNet* and *fpocket* don’t predict residue-level probabilities, AUC and AUPRC could not be reported. F1-scores and Accuracy (ACC) for PUResNet and P2Rank_CONS_ were independently calculated using our evaluation framework, specifically utilizing weighted averaging for F1-scores to better account for class imbalance. The best-performing results are shown in bold.

The results show that pLM finetuning represents a state-of-the-art strategy in residue-level evaluation, as our finetuned models surpass all other reported methods across both datasets. Specifically, the GBS models outperform the second-best reported method by more than 9% in both AUC and AUPRC, and by 7% in MCC (Table 2). Nevertheless, we observe that the application of the pocket smoothing classifier leads to a modest decrease in MCC.

### 3.2 Smoothing and pocket-level evaluation

As summarized in Table 3, pLM finetuning shows improved performance in DCC_top-N_ and DCC_TOP-(N + 2)_ over the fpocket rescored by PRANK (denoted fpocket_PRANK_), setting a new state-of-the-art performance on the LIGYSIS dataset regarding the DCC_top-N_ and DCC_top-(N + 2)_ metrics. In particular, our finetuned approach shows a substantial improvement of 13% in DCC_top-N_ recall and 8% in DCC_top-(N + 2)_ recall compared to previous state-of-the-art (fpocket_PRANK_, P2Rank_CONS_). While Ligsite achieves the highest RRO among the tested methods, its DCC recall remains limited.

**Table 3:** Pocket-level performance comparison across GBS and CBS tasks.

Evaluation	Method	Train set	DCC_top-N_ (%)	DCC_top-(N + 2)_ (%)	DCC_MAX_ (%)	% RRO (%)
CryptoBench test set (CBS)	Seq2Pocket	CryptoBench train	**70.4**	**75.6**	**76.5**	**51.8**
	Seq2Pocket (without smoothing)	CryptoBench train	68.1	73.7	73.7	50.4
	CryptoBench baseline (Škrhák et al. 2025)	CryptoBench train	61.9	73.0	75.4	41.6
LIGYSIS (GBS)	Seq2Pocket	sc-PDB_enhanced_	**62.0**	**68.6**	71.9	49.5
	Seq2Pocket (without smoothing)	sc-PDB_enhanced_	61.3	67.0	69.1	49.5
	Seq2Pocket	sc-PDB	52.6	57.6	59.9	45.3
	PUResNet (Kandel et al. 2021, Utgés and Barton 2024)	sc-PDB	40.6	41.1	41.1	61.0
	P2Rank_CONS_ (Krivák and Hoksza 2015, 2018, Utgés and Barton 2024)	CHEN11	48.8	53.9	57.0	56.4
	fpocket_PRANK_ (Utgés and Barton 2024)	–	48.8	60.4	**91.3**	52.6
	Ligsite (Hendlich et al. 1997, Utgés and Barton 2024]	–	41.3	48.4	49.7	**77.6**
	Only top-performing methods from Utgés and Barton (2024) are shown, but Seq2Pocket (GBS) is implicitly compared against all methods in that study.					

The table compares our proposed finetuning and smoothing approach against state-of-the-art baselines using pocket-level metrics. The top-performing methods in pocket-level metrics from the LIGYSIS study are added, namely fpocket (Le Guilloux *et al.* 2009) and Ligsite (Hendlich *et al.* 1997). The emphasis is on DCC_top-N_ and DCC_top-(N + 2)_, which don’t reward excessive overprediction (i.e. predicting too many binding sites). The best-performing results are shown in bold.

The model trained on the original sc-PDB showed 48.1% DCC_top-(N + 2)_ on small binding sites (less than 10 residues) and 74.7% on large binding sites (10 or more residues). In comparison, after enhancing the training set, the model trained on sc-PDB_enhanced_ achieves 58.9% on small and 81.8% on large binding sites. Therefore, dataset enhancement led to 10% improvement on small binding sites and 7% on large binding sites (see Table S1 in Supplementary Material for more details).

For compatibility with prior literature, we measured the performance of the Seq2Pocket (GBS) model on the COACH420 dataset, where it reaches 90% DCC_top-N_ recall for the 12 Å threshold. Furthermore, we compared scoring the predicted pockets by the sum of squares of residue-level binding probabilities (see Section 2.3) against the simple average of residue-level binding probabilities. On the COACH420 dataset, the sum-of-squares ranking yields a substantial improvement of over 14% in DCC_top-N_ recall relative to the average baseline (see Table S3 in Supplementary Material), while DCC_MAX_ and RRO remain unchanged (as sum-of-squares only affects pocket ordering and doesn’t improve pocket localization).

On LIGYSIS, smoothing adds on average 8.62 residues per structure and although modestly lowers residue-level MCC/ACC/F1 (Table 2), it improves the pocket-level metrics (Table 3, McNemar’s test, *P* < .0001 on the GBS task). Of the residues geometrically inside a pocket’s convex hull but missing from the pre-smoothing prediction, smoothing successfully fills 45% of them. Although it improves the DCC values on the CBS task as well, those results didn’t turn out to be statistically significant (McNemar’s test, *P* = .34).

We utilized P2Rank (Krivák and Hoksza 2018), a state-of-the-art geometry-based predictor. P2Rank is a widely used tool in the LBS task. It identifies binding pockets by analysing the local geometry of the protein surface instead of relying on sequence embeddings. Interestingly, although P2Rank was not trained to reveal cryptic binding sites, it showed competitive performance on the CBS task in multiple studies (Ehrt 2019, Škrhák *et al.* 2024). We run P2Rank on the CryptoBench test set and merged the predictions with the output of the smoothed pLM. The merged prediction achieved DCC_MAX_ greater than 80% and a mean RRO of 57% (see Fig. S7 in Supplementary).

Comparing the Seq2Pocket (CBS) with the CryptoBench baseline reveals significant increase in DCC_top-N_ (Table 3).

To make the models available for hardware settings with more constrained computational resources, we repeated finetuning for the ESM-2 (650M) variant, and compared the performance with the ESM-2 (3B) models (see Table S1 and Fig. S8 in Supplementary Material).

### 3.3 Selecting an optimal clustering strategy

To select the optimal clustering strategy, we evaluated candidate methods based on DCC-based recall and the **Pocket Fragmentation Index** (PFI). An ideal approach identifies binding sites with high localization accuracy (DCC) while maintaining structural integrity through low PFI.

Our analysis performed on the LIGYSIS dataset shows that relying solely on DCC can be misleading. As illustrated in Fig. 3, some methods like DBSCAN (Ester *et al.* 1996) achieve high pocket-level performance (DCC_top-(N + 2)_  =69.2%) only because they fragment a single pocket into numerous small clusters (PFI =4.68). Although adjusting the DBSCAN parameters can reduce this fragmentation, it simultaneously leads to a significant drop in DCC recall (DCC_top-(N + 2)_  =46.4%, PFI =1.46). Our clustering approach, proposed in 2 Methods and Materials, exhibits low fragmentation (PFI =2.05) while maintaining high pocket-level performance (DCC_top-(N + 2)_  =68.6%). All tested configurations are detailed in the Supplementary Materials, Section 1: Tested configurations for clustering.

**Figure 3:**
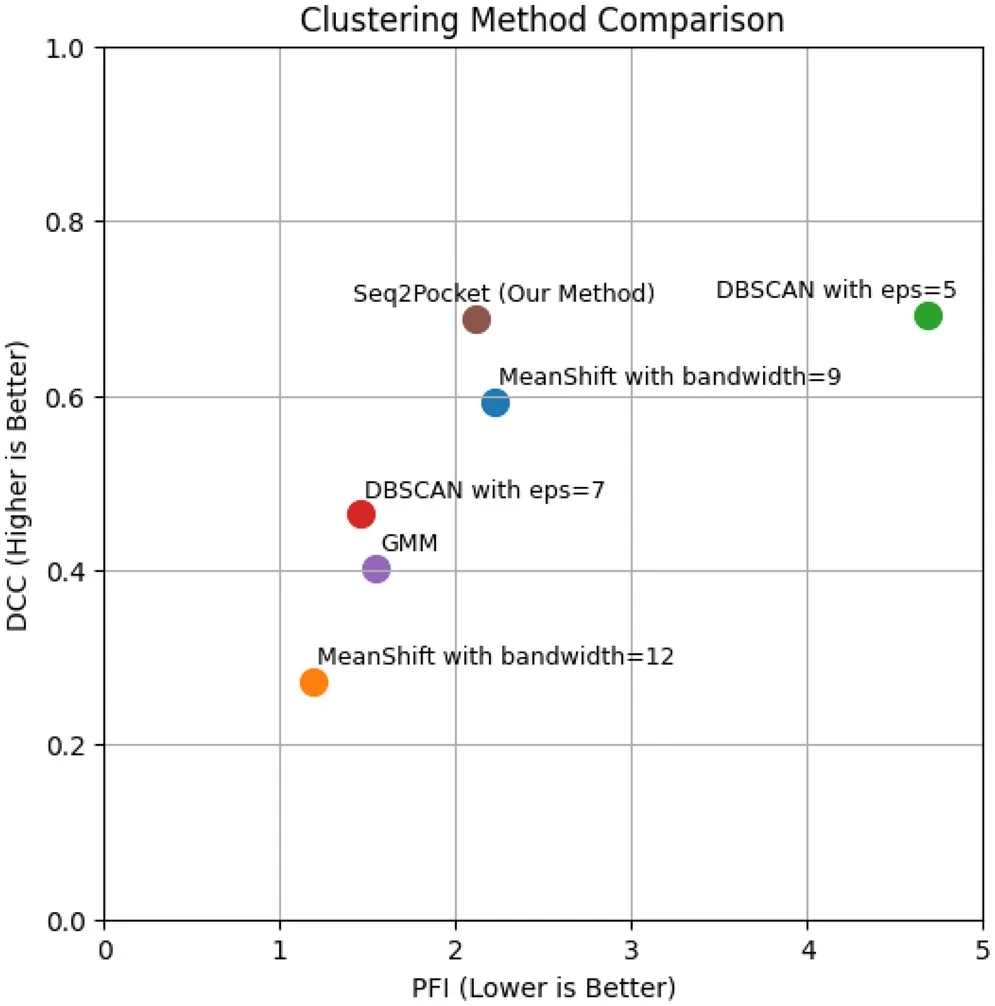
How to select an optimal clustering method. Figure reports the DCC_top-(N + 2)_ metric on the LIGYSIS benchmark. The optimal method should be located in the top-left sector, i.e. showing strong DCC performance while maintaining low fragmentation. All tested configurations and their parameters are detailed in the Supplementary Materials, Section 1.

## 4 Discussion

One of the most interesting observations made during the development of the Seq2Pocket model is that the performance of LBS predictors is strongly influenced by factors beyond the core model architecture. In particular, we show that **training data** and **post-processing** play an important role in binding site prediction performance.

### 4.1 Bias towards large binding sites

Current benchmarks and datasets are heavily biased toward large, orthosteric binding sites, which are overrepresented in datasets such as sc-PDB. As a result, many predictors are underperforming on smaller, less obvious sites. This limitation is not primarily a modeling issue, but rather a consequence of incomplete training data. Many datasets frequently include only the main ligand binding sites (often just the primary orthosteric site) and ignore additional small-molecule or ion-binding events, or binding sites observed in alternative conformations of the same protein.

We reduce this bias by constructing the **sc-PDB_enhanced_** dataset and training our model on it, which incorporates all experimentally observed binding sites including ions. The resulting model achieves improvements across all evaluated metrics, outperforming the model trained on the original sc-PDB by more than 5% in AUPRC, and 9% in DCC_top-N_ at the pocket level (see Table 3). These results indicate that augmenting training data with diverse binding conformations is essential for improving performance on non-obvious targets in our benchmarks. Although previously, DL-based approaches were rendered inefficient when compared with existing geometry-based approaches like fpocket or P2Rank (Utgés and Barton 2024), we argue that this limitation might originate from suboptimal training data rather than from suboptimal model architecture. On top of the sequence-level filtering control (Table S4), we additionally split the LIGYSIS evaluation set by the structural similarity of each test pocket to its closest match in sc-PDB_enhanced_ [Foldseek (Kempen *et al.* 2023) prescreen followed by ProBiS (Konc and Janežič 2010) local structural alignment, Table S5]. Performance on test pockets with no structurally similar training-set counterpart is only modestly lower (∼6% in DCC_MAX_) than on the full evaluation set, indicating that the model’s performance is not driven by memorization of specific training-pocket motifs.

We also note that while traditional datasets like sc-PDB focus solely on organic drug-like ligands (Kellenberger *et al.* 2006, Meslamani *et al.* 2011, Desaphy *et al.* 2015, Cimermancic *et al.* 2016, Meller *et al.* 2023, Škrhák *et al.* 2024, Utgés and Barton 2024], LIGYSIS also treats ions as valid binding molecules. There is no universal “correct” definition; rather, the inclusion of ions depends on the specific downstream application. However, this needs to be taken into account when collecting training data. As demonstrated in Table 3, the improvement from enhancing the dataset is significant (i.e. $>$9% in DCC). Therefore, we strongly encourage developers of binding site prediction methods not only to rely on the pre-existing binding site datasets but also to screen other available protein structures (Baskaran 2018) for additional binding sites that might be included in their training sets, e.g. through sequence and structure similarity.

### 4.2 Metrics inflation through clustering fragmentation

Translating probabilities into meaningful 3D pockets is sensitive to the clustering strategy. We demonstrate that high pocket-level recall, particularly DCC, can be artificially inflated through **clustering fragmentation**, where a single functional binding region is partitioned into multiple small predicted pockets. While this fragmentation increases the chance that at least one cluster lies close to the pocket center, it does not improve the identification of binding sites and substantially reduces the practical usability of the predictions.

We therefore define **Pocket Fragmentation Index**, which measures the correspondence between predicted and actual pockets. Some methods such as DBSCAN often achieve high recall by producing severely fragmented outputs (PFI >4). Our proposed pipeline maintains a reasonable balance between pocket-level performance and binding site integrity.

### 4.3 Spatial inconsistency

To recover residues initially missed by the finetuned pLM during prediction (as illustrated in Figs. 1, 2 and Figs. S2, S3 in the Supplementary Material), we apply a post-processing step (*smoothing*) that leverages surrounding-residue context. Smoothing improves pocket completeness: of the residues that belong to a pocket’s convex hull but were omitted by the pLM prediction, smoothing adds 45% of them. Furthermore, it improves pocket-level metrics on the GBS task, although the improvement on the CBS task is not statistically significant, likely due to its smaller sample size.

### 4.4 Selection of ranking method

In a typical scenario, a user would expect a binding site prediction method to rank large, confident binding sites above smaller ones. Datasets such as LIGYSIS, however, weigh large and small binding sites equally, so a method that violates this expectation—e.g. ranking a small but confident site above a large one—is not penalized, and the problem can go unnoticed. This limitation becomes apparent only when the method is evaluated on datasets predominantly composed of large (often orthosteric) binding sites, such as COACH420. Although we consider LIGYSIS the more appropriate dataset for evaluating binding site prediction methods, we recommend also evaluating on datasets such as COACH420 to capture this kind of behavior.

### 4.5 Implications for evaluation practice

Taken together, our results show that strong residue-level performance does not guarantee accurate pocket-level predictions. Several recent studies report only residue-level metrics and omit pocket-level evaluation altogether (Littmann *et al.* 2021, Wang *et al.* 2024, Mou *et al.* 2025), which can misrepresent actual pocket-level performance. As demonstrated by the disagreement between Tables 2 and 3, evaluating both levels is essential. Furthermore, we recommend controlling for artificial performance inflation at the pocket level, e.g. by reporting fragmentation-aware measures such as the proposed Pocket Fragmentation Index (PFI).

## 5 Conclusion

Protein language models are becoming essential in computational biology pipelines. This work addresses the challenge of transitioning from sequence-level pLM predictions to 3D structural pockets. We show that training data quality is a major driver of this performance: by extending sc-PDB with additional experimentally observed binding sites into sc-PDB_enhanced_, we improved the performance of the model significantly. We complement this with post-processing steps, a smoothing classifier and a clustering strategy, that address the fragmentation of sequence-level predictions. Together, our framework achieves the best DCC_top-N_ and DCC_top-(N + 2)_ among the compared methods on both benchmarks: CryptoBench for cryptic pocket prediction and LIGYSIS for general pocket detection. We believe that our insights, particularly on the importance of training data curation, will stimulate further development in the binding site prediction domain.

## Supplementary Material

btag778_Supplementary_Data

## Data Availability

The Seq2Pocket web interface running the ESM-2 650M variant is available at https://seq2pocket.projekty.ms.mff.cuni.cz. The code created for the purpose of this manuscript can be found at https://github.com/skrhakv/seq2pocket. The repository also includes a short tutorial for running the proposed methodology. The sc-PDB_enhanced_ is published using Zenodo platform at https://zenodo.org/records/18271517 (Škrhák and Hoksza 2026). The trained models can be downloaded at https://s3.cl4.du.cesnet.cz/a93fcece52e6da0dd335b4459d47b0aebb74836b: share/seq2pocket-models.tar.gz. Dataset annotations, and prepared PyMOL scripts for visualization of the predictions can be downloaded at https://s3.cl4.du.cesnet.cz/a93fcece52e6da0dd335b4459d47b0aebb74836b: share/seq2pocket-data.tar.gz. Lastly, the models are also published at HuggingFace: https://huggingface.co/skrhakv/seq2pocket.

## References

[btag778-B1] Abraham MJ , Murtola T, Schulz R et al Gromacs: high performance molecular simulations through multi-level parallelism from laptops to supercomputers. SoftwareX 2015;1–2:19–25. doi: 10.1016/j.softx.2015.06.001.

[btag778-B2] Aggarwal R , Gupta A, Chelur V et al Deeppocket: ligand binding site detection and segmentation using 3d convolutional neural networks. J Chem Inf Model 2022;62:5069–79. 10.1021/acs.jcim.1c00799 PMID: 34374539. 34374539

[btag778-B3] Burley SK , Berman HM, Bhikadiya C et al Protein Data Bank: the single global archive for 3D macromolecular structure data. Nucleic Acids Res 2019;47:D520–8. 10.1093/nar/gky949 PMC6324056 30357364

[btag778-B4] Beglov D , Hall DR, Wakefield AE et al Exploring the structural origins of cryptic sites on proteins. Proc Natl Acad Sci USA 2018;115:E3416–E3425. 10.1073/pnas.1711490115 PMC5899430 29581267

[btag778-B5] Brylinski M , Skolnick J. What is the relationship between the global structures of apo and holo proteins? Proteins Struct Funct Bioinf 2008;70:363–77. 10.1002/prot.21510. 17680687

[btag778-B6] Carbery A , Buttenschoen M, Skyner R et al Learnt representations of proteins can be used for accurate prediction of small molecule binding sites on experimentally determined and predicted protein structures. J Cheminform 2024;16:32. 10.1186/s13321-024-00821-4 PMC10941399 38486231

[btag778-B7] Cheng Y. Mean shift, mode seeking, and clustering. IEEE Trans Pattern Anal Mach Intell 1995;17:790–9. 10.1109/34.400568

[btag778-B8] Chicco D , Jurman G. The advantages of the Matthews correlation coefficient (mcc) over f1 score and accuracy in binary classification evaluation. BMC Genomics 2020;21:6. 10.1186/s12864-019-6413-7 PMC6941312 31898477

[btag778-B9] Cimermancic P , Weinkam P, Rettenmaier TJ et al Cryptosite: expanding the druggable proteome by characterization and prediction of cryptic binding sites. J Mol Biol 2016;428:709–19. 10.1016/j.jmb.2016.01.029 PMC4794384 26854760

[btag778-B10] Cock PJA , Antao T, Chang JT et al Biopython: freely available python tools for computational molecular biology and bioinformatics. Bioinformatics 2009;25:1422–3. 10.1093/bioinformatics/btp163 PMC2682512 19304878

[btag778-B11] De Vivo M , Masetti M, Bottegoni G et al Role of molecular dynamics and related methods in drug discovery. J Med Chem 2016;59:4035–61. 10.1021/acs.jmedchem.5b01684 26807648

[btag778-B12] Desaphy J , Bret G, Rognan D et al Sc-PDB: a 3D-database of ligandable binding sites-10 years on. Nucleic Acids Res 2015;43:D399–404. 10.1093/nar/gku928 PMC4384012 25300483

[btag778-B13] Ehrt C. *Protein binding site comparison*. Phd Thesis, Technische Universität Dortmund, 2019.

[btag778-B14] Elnaggar A , Heinzinger M, Dallago C et al Prottrans: toward understanding the language of life through self-supervised learning. IEEE Trans Pattern Anal Mach Intell 2022;44:7112–27. 10.1109/TPAMI.2021.3095381 34232869

[btag778-B15] Ester M , Kriegel H-P, Sander J et al A density-based algorithm for discovering clusters in large spatial databases with noise. In *Proceedings of the Second International Conference on Knowledge Discovery and Data Mining*, KDD’96, pp. 226–231. AAAI Press, 1996.

[btag778-B16] Feidakis CP , Krivak R, Hoksza D et al Ahoj: rapid, tailored search and retrieval of apo and holo protein structures for user-defined ligands. Bioinformatics 2022;38:5452–3. 10.1093/bioinformatics/btac701 PMC9750100 36282546

[btag778-B17] Feidakis CP , Krivak R, Hoksza D et al Ahoj-db: a pdb-wide assignment of apo & holo relationships based on individual protein–ligand interactions. J Mol Biol 2024;436:168545. 10.1016/j.jmb.2024.168545 38508305

[btag778-B18] Ferreira L , Santos R, Oliva G et al Molecular docking and structure-based drug design strategies. Molecules 2015;20:13384–421. 10.3390/molecules200713384 PMC6332083 26205061

[btag778-B19] Gamouh H , Novotný M, Hoksza D. Hybrid protein–ligand binding residue prediction with protein language models: does the structure matter? Bioinformatics 2025;41:btaf431. 10.1093/bioinformatics/btaf431 PMC12377911 40742755

[btag778-B20] Gašparíková D , Chikhale R, Cole J et al Recent computational advances in the identification of cryptic binding sites for drug discovery. Bioinform Adv 2025;5:vbaf156. 10.1093/bioadv/vbaf156 PMC12342141 40799497

[btag778-B21] Halgren TA. Identifying and characterizing binding sites and assessing druggability. J Chem Inf Model 2009;49:377–89. 10.1021/ci800324m 19434839

[btag778-B22] Heinzinger M , Elnaggar A, Wang Y et al Modeling aspects of the language of life through transfer-learning protein sequences. BMC Bioinformatics 2019;20:723. 10.1186/s12859-019-3220-8 PMC6918593 31847804

[btag778-B23] Heinzinger M , Weissenow K, Sanchez J et al Bilingual language model for protein sequence and structure. NAR Genom Bioinform 2024;6:lqae150. 10.1093/nargab/lqae150 PMC11616678 39633723

[btag778-B24] Hendlich M , Rippmann F, Barnickel G. Ligsite: automatic and efficient detection of potential small molecule-binding sites in proteins. J Mol Graph Model 1997;15:359–63. 10.1016/s1093-3263(98)00002-3 9704298

[btag778-B25] Jiménez-Luna J , Grisoni F, Weskamp N et al Artificial intelligence in drug discovery: recent advances and future perspectives. Expert Opin Drug Discov 2021;16:949–59. 10.1080/17460441.2021.1909567 33779453

[btag778-B26] Jumper J , Evans R, Pritzel A et al Highly accurate protein structure prediction with alphafold. Nature 2021;596:583–9. 10.1038/s41586-021-03819-2 PMC8371605 34265844

[btag778-B27] Kandel J , Tayara H, Chong K. Puresnet: prediction of protein–ligand binding sites using deep residual neural network. J Cheminform 2021;13:65. 10.1186/s13321-021-00547-7 PMC8424938 34496970

[btag778-B28] Jeevan K , Palistha S, Tayara H et al Puresnetv2.0: a deep learning model leveraging sparse representation for improved ligand binding site prediction. J Cheminform 2024;16:66. 10.1186/s13321-024-00865-6 PMC11157904 38849917

[btag778-B29] Kellenberger E , Muller P, Schalon C et al sc-pdb: an annotated database of druggable binding sites from the protein data bank. J Chem Inf Model 2006;46:717–27. 10.1021/ci050372x 16563002

[btag778-B31] Konc J , Janežič D. Probis algorithm for detection of structurally similar protein binding sites by local structural alignment. Bioinformatics 2010;26:1160–8. 10.1093/bioinformatics/btq100 PMC2859123 20305268

[btag778-B32] Kozakov D , Grove L, Hall D et al The ftmap family of web servers for determining and characterizing ligand-binding hot spots of proteins. Nat Protoc 2015;10:733–55. 10.1038/nprot.2015.043 PMC4762777 25855957

[btag778-B33] Krivák R , Hoksza D. P2rank: machine learning based tool for rapid and accurate prediction of ligand binding sites from protein structure. J Cheminform 2018;10:39. 10.1186/s13321-018-0285-8 PMC6091426 30109435

[btag778-B34] Krivák R , Hoksza D. P2rank: knowledge-based ligand binding site prediction using aggregated local features. In Algorithms for Computational Biology. Cham: Springer International Publishing, 2015, 41–52.

[btag778-B35] Lamers MB , Antson AA, Hubbard RE et al Structure of the protein tyrosine kinase domain of c-terminal src kinase (csk) in complex with staurosporine11edited by I. A. Wilson. J Mol Biol 1999;285:713–25. 10.1006/jmbi.1998.2369. 9878439

[btag778-B36] Laurie A , Jackson R. Q-sitefinder: an energy-based method for the prediction of protein–ligand binding sites. Bioinformatics 2005;21:1908–16. 10.1093/bioinformatics/bti315 15701681

[btag778-B37] Le Guilloux V , Schmidtke P, Tuffery P. Fpocket: an open source platform for ligand pocket detection. BMC Bioinformatics 2009;10:168. 10.1186/1471-2105-10-168 PMC2700099 19486540

[btag778-B38] Li Y , Huang C, Ding L et al Deep learning in bioinformatics: introduction, application, and perspective in the big data era. Methods 2019;166:4–21. 10.1016/j.ymeth.2019.04.008 31022451

[btag778-B39] Lin Z , Akin H, Rao R et al Evolutionary-scale prediction of atomic-level protein structure with a language model. Science 2023;379:1123–30. 10.1126/science.ade2574 36927031

[btag778-B40] Littmann M , Heinzinger M, Dallago C et al Protein embeddings and deep learning predict binding residues for various ligand classes. Sci Rep 2021;11:23916. 10.1038/s41598-021-03431-4 PMC8668950 34903827

[btag778-B41] Meller A , Ward M, Borowsky J et al Predicting locations of cryptic pockets from single protein structures using the pocketminer graph neural network. Nat Commun 2023;14:1177. 10.1038/s41467-023-36699-3 PMC9977097 36859488

[btag778-B42] Meslamani J , Rognan D, Kellenberger E. sc-pdb: a database for identifying variations and multiplicity of ‘druggable’ binding sites in proteins. Bioinformatics 2011;27:1324–6. 10.1093/bioinformatics/btr120 21398668

[btag778-B43] Mou M , Lu M, Zhou Z et al Accurate identification of protein binding sites for all drug modalities using allsites. Adv Sci (Weinh) 2026;13:e16530. 10.1002/advs.202516530. PMC12915145 41454719

[btag778-B44] Pugh CWJ , Nuñez-Valencia PG, Dias M et al From likelihood to fitness: improving variant effect prediction in protein and genome language models. bioRxiv, 10.1101/2025.05.20.655154, 2025, preprint: not peer reviewed.

[btag778-B45] Roy A , Yang J, Zhang Y. Cofactor: an accurate comparative algorithm for structure-based protein function annotation. Nucleic Acids Res 2012;40:W471–7. 10.1093/nar/gks372 PMC3394312 22570420

[btag778-B46] Sestak F , Schneckenreiter L, Brandstetter J et al Vn-egnn: E(3)-equivariant graph neural networks with virtual nodes enhance protein binding site identification. 2024. 10.1186/s13321-025-01127-9 PMC12837241 41398608

[btag778-B47] Shrake A , Rupley J. Environment and exposure to solvent of protein atoms. lysozyme and insulin. J Mol Biol 1973;79:351–71. 10.1016/0022-2836(73)90011-9 4760134

[btag778-B48] Škrhák V , Hoksza D. scpdb-enhanced: An expansion of scpdb including all experimentally observed binding sites. 2026.

[btag778-B49] Škrhák V , Gamouh H, Hoksza D. Hidden in Protein Sequences: Predicting Cryptic Binding Sites. New York: Association for Computing Machinery, 2025.

[btag778-B50] Škrhák V , Novotný M, Feidakis CP et al Cryptobench: cryptic protein–ligand binding sites dataset and benchmark. Bioinformatics 2024;41:btae745. 10.1093/bioinformatics/btae745 PMC11725321 39693053

[btag778-B51] Škrhák V , Riedlova K, Novotny M et al Cryptic binding site prediction with protein language models. In *2023 IEEE International Conference on Bioinformatics and Biomedicine (BIBM)*, pp. 2883–2888, Los Alamitos, CA, USA. IEEE Computer Society, 2023. 10.1109/BIBM58861.2023.10385497

[btag778-B52] Smith Z , Strobel M, Vani BP et al Graph attention site prediction (grasp): identifying druggable binding sites using graph neural networks with attention. J Chem Inf Model 2024;64:2637–44. 10.1021/acs.jcim.3c01698 PMC11182664 38453912

[btag778-B53] Southey M , Brunavs M. Introduction to small molecule drug discovery and preclinical development. Front Drug Discov 2023;3:11. 10.3389/fddsv.2023.1314077

[btag778-B54] Srivastava N , Hinton G, Krizhevsky A et al Dropout: a simple way to prevent neural networks from overfitting. J Mach Learn Res 2014;15:1929–58. http://jmlr.org/papers/v15/srivastava14a.html

[btag778-B55] Steinegger M , Söding J. Mmseqs2 enables sensitive protein sequence searching for the analysis of massive data sets. Nat Biotechnol 2017;35:1026–8. 10.1038/nbt.3988 29035372

[btag778-B56] Utgés JS. Lbs-comparison results. 2024.

[btag778-B57] Utgés J , Barton G. Comparative evaluation of methods for the prediction of protein–ligand binding sites. J Cheminform 2024;16:126. 10.1186/s13321-024-00923-z PMC11552181 39529176

[btag778-B58] Vajda S , Beglov D, Wakefield AE et al Cryptic binding sites on proteins: definition, detection, and druggability. Curr Opin Chem Biol 2018;44:1–8. 10.1016/j.cbpa.2018.05.003 PMC6088748 29800865

[btag778-B30] Van Kempen M , Kim SS, Tumescheit C et al Fast and accurate protein structure search with Foldseek. Nat Biotechnol 2024;42:243–6. 10.1038/s41587-023-01773-0 PMC10869269 37156916

[btag778-B59] Wang B , Geng Y, Cheng X et al Protgo: universal protein function prediction utilizing multi-modal gene ontology knowledge. Bioinformatics 2025a;41:btaf390. 10.1093/bioinformatics/btaf390 PMC12296341 40632605

[btag778-B60] Wang J , Liu Y, Tian B. Protein-small molecule binding site prediction based on a pre-trained protein language model with contrastive learning. J Cheminform 2024;16:125. 10.1186/s13321-024-00920-2 PMC11542454 39506806

[btag778-B61] Wang W , Liang P-Y, Bai J. DeepPH: A Multimodal Deep Learning Model for Predicting Enzyme Optimal pH Range. New York: Association for Computing Machinery, 2025b.

[btag778-B62] Yang J , Roy A, Zhang Y. Protein–ligand binding site recognition using complementary binding-specific substructure comparison and sequence profile alignment. Bioinformatics 2013;29:2588–95. 10.1093/bioinformatics/btt447 PMC3789548 23975762

[btag778-B63] Zhang Z , Wayment-Steele HK, Brixi G et al Protein language models learn evolutionary statistics of interacting sequence motifs. Proc Natl Acad Sci USA 2024;121:e2406285121. 10.1073/pnas.2406285121 PMC11551344 39467119

[btag778-B64] Zhao J , Cao Y, Zhang L. Exploring the computational methods for protein–ligand binding site prediction. Comput Struct Biotechnol J 2020;18:417–26. 10.1016/j.csbj.2020.02.008 PMC7049599 32140203

