# Supplementary material for: Seq2Pocket: augmenting protein language models for spatially consistent binding site prediction
Source: Bioinformatics. 2026 Oct 16;42(10):btag778. doi: 10.1093/bioinformatics/btag778 (PMC13712190; doi:10.1093/bioinformatics/btag778)
Supplement: Supplementary file 1 [file btag778_supplementary_data.pdf]

Supporting Information for

Seq2Pocket: Augmenting protein language models for spatially consistent binding site prediction

Vít Škrhák, Lukáš Polák, Marian Novotný and David Hoksza

Corresponding Author: Vít Škrhák.
E-mail: vit.skrhak@matfyz.cuni.cz

This PDF file includes:

Supporting text
Figs. S1 to S8
Tables S1 to S5
SI References

Supporting Information Text

1. Tested configurations for clustering

Here we describe the three alternative clustering configurations that, together with the primary SAS-point method, were the candidates we selected from when choosing the final clustering procedure. Although our primary approach clusters Solvent Accessible Surface (SAS) points, these alternative configurations operate by clustering residues. We utilized several algorithms from the *scikit-learn* library, specifically DBSCAN ($eps \in \{5, 7\}$, where eps is maximum distance between two points to be considered part of a single cluster), MeanShift ($bandwidth \in \{9, 12\}$), and Bayesian Gaussian Mixture Modeling (GMM). The evaluation workflow was structured as follows:

- Extraction of predicted binding residues using pLM.
- Clustering of identified residues using one of the candidate algorithms.
- Application of a smoothing classifier to refine the spatial boundaries of the predicted pockets.

We note that this order differs slightly from the main clustering configuration (SAS-point-based configuration), where the smoothing is applied first and clustering second.

Although the implementation of DBSCAN and MeanShift followed standard procedures (i.e., initializing the sklearn algorithm object and executing $fit\_predict$ method), the Bayesian Gaussian Mixture Modeling (**GMM**) approach required a two-stage process:

1. **Cluster Estimation:** An initial GMM run was performed to estimate the optimal number of clusters. A cluster was considered significant if it contained at least 10% of the total predicted binding residues.
2. **Final Clustering:** The Bayesian GMM was re-initialized and executed using the number of significant clusters determined in the first stage.

2. sc-PDB$_{\text{enhanced}}$ construction protocol

A. AHoJ-DB background. AHoJ-DB (1, 2) is a PDB-wide database built on top of the AHoJ tool. For each annotated ligand-bound (holo) interaction in the PDB, AHoJ identifies the binding-site residues in contact with the ligand within a fixed distance cutoff (4.5 Å). It then retrieves every other PDB chain sharing the same UniProt accession, aligns each of them to the query chain (TM-align), maps the query binding-site residues onto the aligned chain, and classifies the aligned chain as holo if a compatible ligand occupies the mapped site, or apo if it does not. Applied across the whole PDB, this produces, for a large fraction of proteins, a set of alternative structural conformations and ligand-occupancy states per binding site, including sites and ligands (e.g. ions, small buffer-scale ligands) absent from curated single-ligand-per-entry resources such as sc-PDB. sc-PDB$_{\text{enhanced}}$ reuses these AHoJ-DB pocket-residue annotations to augment sc-PDB's original site labels.

B. Structure/pair selection. From the AHoJ-DB v2c batch output, an entry is retained for the enhancement step only if:

- AHoJ-DB produced a complete result for it (query pocket info, pocket-residue selections, and apo-search results all present);
- the holo structure is single-chain (multi-chain holo entries are excluded at this stage);
- the holo structure corresponds to a single UniProt accession (hetero-oligomeric complexes are excluded).

C. Merging with sc-PDB$_{\text{enhanced}}$. AHoJ-DB entries are matched to sc-PDB v2017 by PDB ID and author-provided (auth) chain identifier. For every sc-PDB chain, the residues from all matched AHoJ-DB pocket annotations are combined with the original sc-PDB binding-site residues for that chain into a single union set. This union, i.e. one binding-residue set per chain, constitutes the sc-PDB$_{\text{enhanced}}$ labels used for residue-level model training.

D. Train/test leakage removal. Each chain in sc-PDB$_{\text{enhanced}}$ is mapped to a UniProt accession via the PDBe API. Any chain whose UniProt accession also occurs in the LIGYSIS evaluation set is removed from the training data. This filtering reduces the number of training structures from 17,077 to 13,140 (Table 1), and is applied identically to both sc-PDB and sc-PDB$_{\text{enhanced}}$ to keep the two training sets directly comparable.

E. Code availability. The scripts are available in the CryptoBench repository, branch scPDB_enhancement (src/A-filter-ahojdb/): *https://github.com/skrhakv/CryptoBench/tree/scPDB_enhancement*

3. Hyperparameter Selection

The residue-level pLM decision threshold (0.70) was selected by scanning thresholds in {0.10, 0.15, ..., 0.90} and choosing the value maximizing MCC on a held-out validation split (194 proteins) of the CryptoBench training set.

The smoothing classifier's hidden dimension, dropout rate, and learning rate were selected via grid search over {256, 512, 1024, 2048} x {0.1, 0.3, 0.5} x {1e-5, 1e-4, 1e-3}, maximizing AUPRC on the same validation split; the batch size was then selected from {256, 512, 1024, 2048, 4096} using the same criterion. This yielded hidden dim = 2048, dropout = 0.5, learning rate = 1e-4, batch size = 512.

The smoothing decision threshold (0.40) was selected by scanning thresholds in {0.1, 0.2, ..., 0.9} and choosing the value maximizing MCC on the same validation split.

The learning rate for the finetuned model was selected via search over {1e-5, 1e-4, 1e-3}, maximizing AUPRC on the same validation split. The number of epochs was selected from the {2,3,4} span.

The MeanShift bandwidth (9 Å) was selected from {6, 7, 9, 12, 15} A on the CryptoBench validation split, evaluating DCC top-(N+2).

The SAS probe radius (1.6 Å) was selected from {1.4, 1.6, 2.0} A on the same validation split. DCC top-(N+2) was the same for 1.6 Å and 2.0 Å, therefore PFI was selected as a secondary metric to select 1.6 Å.

The surface point density (50 points/atom) was selected by comparing {20, 50, 100} on the same validation split. DCC top-(N+2) was comparable across the grid (±0.005); 20 was excluded due to higher PFI and 100 for its higher runtime cost (avg. 0.66 seconds/protein for 100 points/atom, 0.52 seconds/protein for 50 points/atom).

The candidate radius of 15 Å and negative-sample radius of 10 Å were both selected from the {10, 15} values based on the AUPRC metric.

4. Selection of methods for comparison

For the GBS task, we adopt the same set of comparison methods as Utgés et al. (3), as their benchmark provides a well-documented and reproducible basis for evaluating binding site predictors on the LIGYSIS dataset. Several recently proposed tools, including EquiPocket, were already excluded from that benchmark due to missing or incomplete documentation and installation instructions (see Supplementary Table 1 in Utgés et al. (3)). We additionally evaluated AF2Bind (4), published after Utgés et al., but were unable to reproduce the results reported in the original study using the guidelines provided in its GitHub repository. We therefore excluded AF2Bind from our comparison on grounds of limited reproducibility.

References

1. Christos P Feidakis, Radoslav Krivak, David Hoksza, and Marian Novotny. Ahoj: rapid, tailored search and retrieval of apo and holo protein structures for user-defined ligands. *Bioinformatics*, 38(24):5452–5453, 10 2022. ISSN 1367-4811. . URL https://doi.org/10.1093/bioinformatics/btac701.
2. Christos P. Feidakis, Radoslav Krivak, David Hoksza, and Marian Novotny. Ahoj-db: A pdb-wide assignment of apo & holo relationships based on individual protein–ligand interactions. *Journal of Molecular Biology*, 436(17):168545, 2024. ISSN 0022-2836. . URL https://www.sciencedirect.com/science/article/pii/S0022283624001402. Computation Resources for Molecular Biology.
3. Javier Utgés and Geoffrey Barton. Comparative evaluation of methods for the prediction of protein–ligand binding sites. *Journal of Cheminformatics*, 16, 11 2024. .
4. Artem Gazizov, Anna Lian, Casper Goverde, Jody Mou, Sergey Ovchinnikov, and Nicholas Polizzi. Af2bind: predicting small-molecule binding sites using the pair representation of alphafold2. *Nature methods*, 23, 03 2026. .
5. Yizong Cheng. Mean shift, mode seeking, and clustering. *IEEE Transactions on Pattern Analysis and Machine Intelligence*, 17(8):790–799, 1995. .
6. Vít Škrhák, Marian Novotný, Christos P Feidakis, Radoslav Krivák, and David Hoksza. Cryptobench: cryptic protein–ligand binding sites dataset and benchmark. *Bioinformatics*, 41(1):btae745, 12 2024. ISSN 1367-4811. . URL https://doi.org/10.1093/bioinformatics/btae745.
7. Jamel Meslamani, Didier Rognan, and Esther Kellenberger. sc-pdb: a database for identifying variations and multiplicity of 'druggable' binding sites in proteins. *Bioinformatics*, 27(9):1324–1326, 03 2011. ISSN 1367-4803. . URL https://doi.org/10.1093/bioinformatics/btr120.
8. Jérémy Desaphy, Guillaume Bret, Didier Rognan, and Esther Kellenberger. sc-pdb: a 3d-database of ligandable binding sites—10 years on. *Nucleic Acids Research*, 43(D1):D399–D404, 10 2014. ISSN 0305-1048. . URL https://doi.org/10.1093/nar/gku928.
9. Radoslav Krivák and David Hoksza. P2rank: machine learning based tool for rapid and accurate prediction of ligand binding sites from protein structure. *Journal of cheminformatics*, 10:1–12, 2018.
10. Michel Kempen, Stephanie Kim, Charlotte Tumescheit, Milot Mirdita, Jeongjae Lee, Cameron Gilchrist, Johannes Söding, and Martin Steinegger. Fast and accurate protein structure search with foldseek. *Nature Biotechnology*, 42:1–4, 05 2023. .
11. Janez Konc and Dušanka Janežič. Probis algorithm for detection of structurally similar protein binding sites by local structural alignment. *Bioinformatics*, 26(9):1160–1168, 05 2010. ISSN 1367-4803. . URL https://doi.org/10.1093/bioinformatics/btq100.

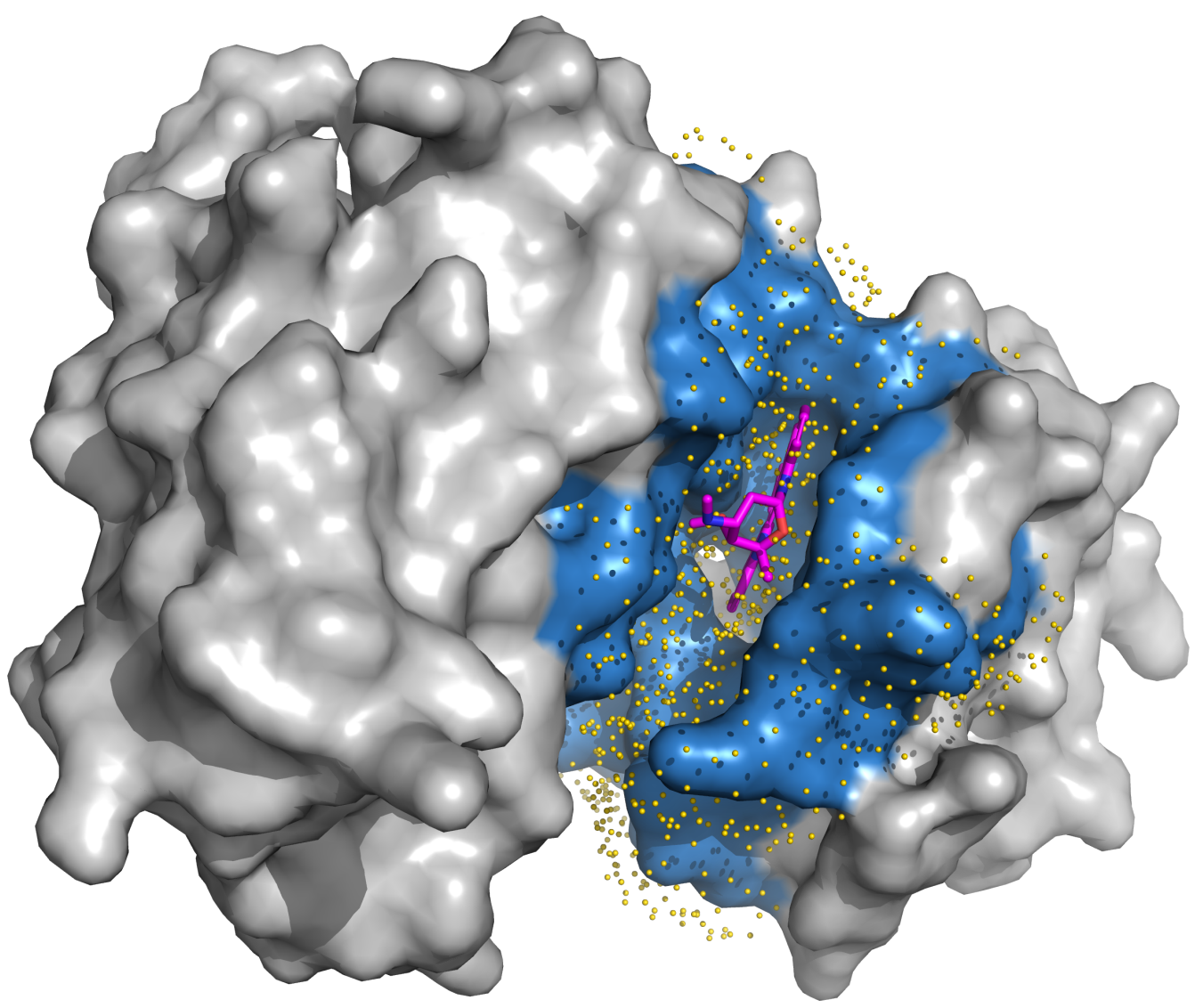

Fig. S1. SAS points on predicted residues. Here, the fine-tuned GBS-pLM model correctly predicts a binding site on the human C-terminal Src kinase (*PDB ID: 1BYG, chain A*), where an inhibitor Staurosporine binds. The yellow dots represent the solvent accessible surface (**SAS**) points, that are subsequently clustered using MeanShift algorithm (5).

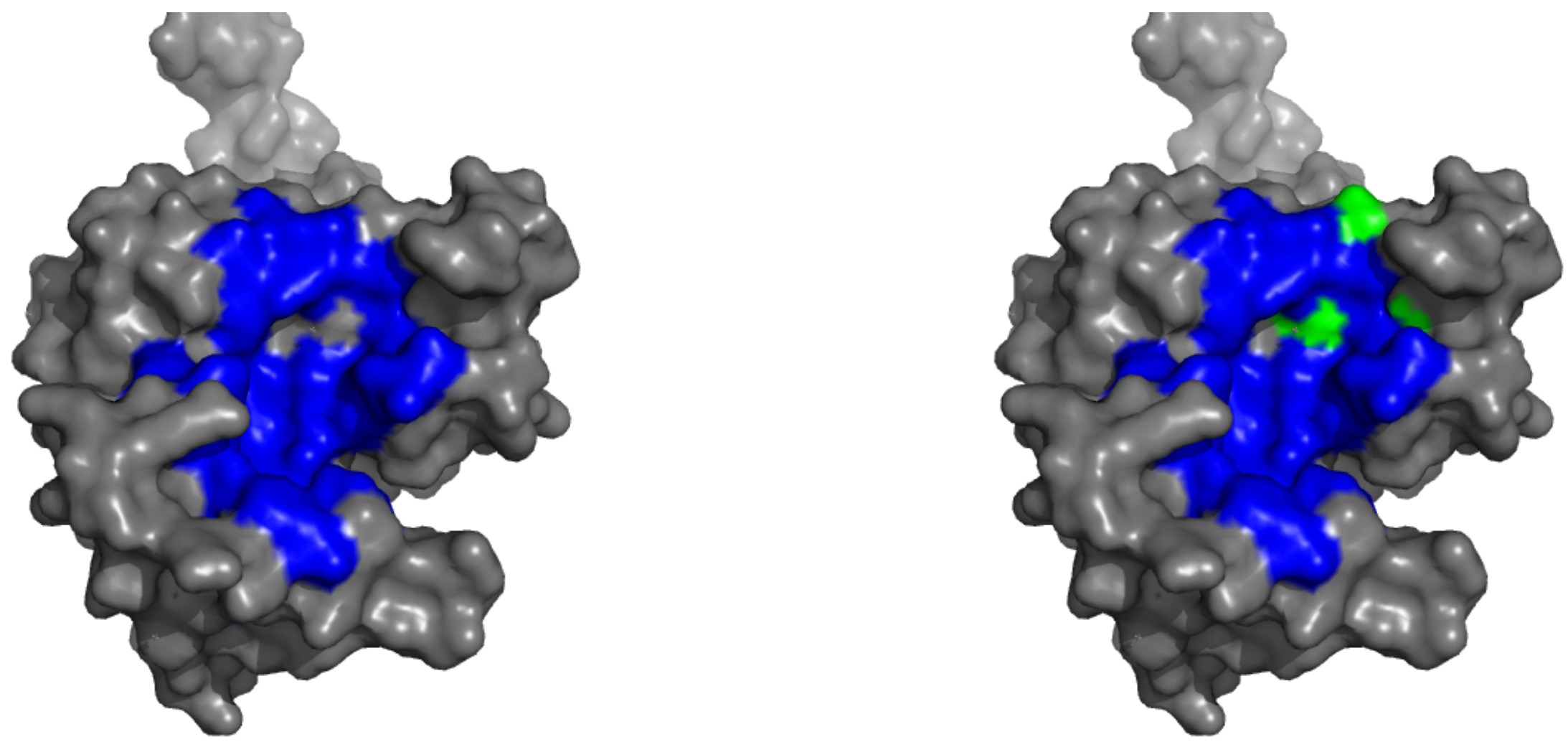

(a) Without smoothing

(b) With smoothing

Fig. S2. Example of residues added by smoothing classifier (GREEN: Thr213, Asp216, Leu218) of a predicted pocket (BLUE). This is a 7nlxA structure from the CryptoBench test set (6).

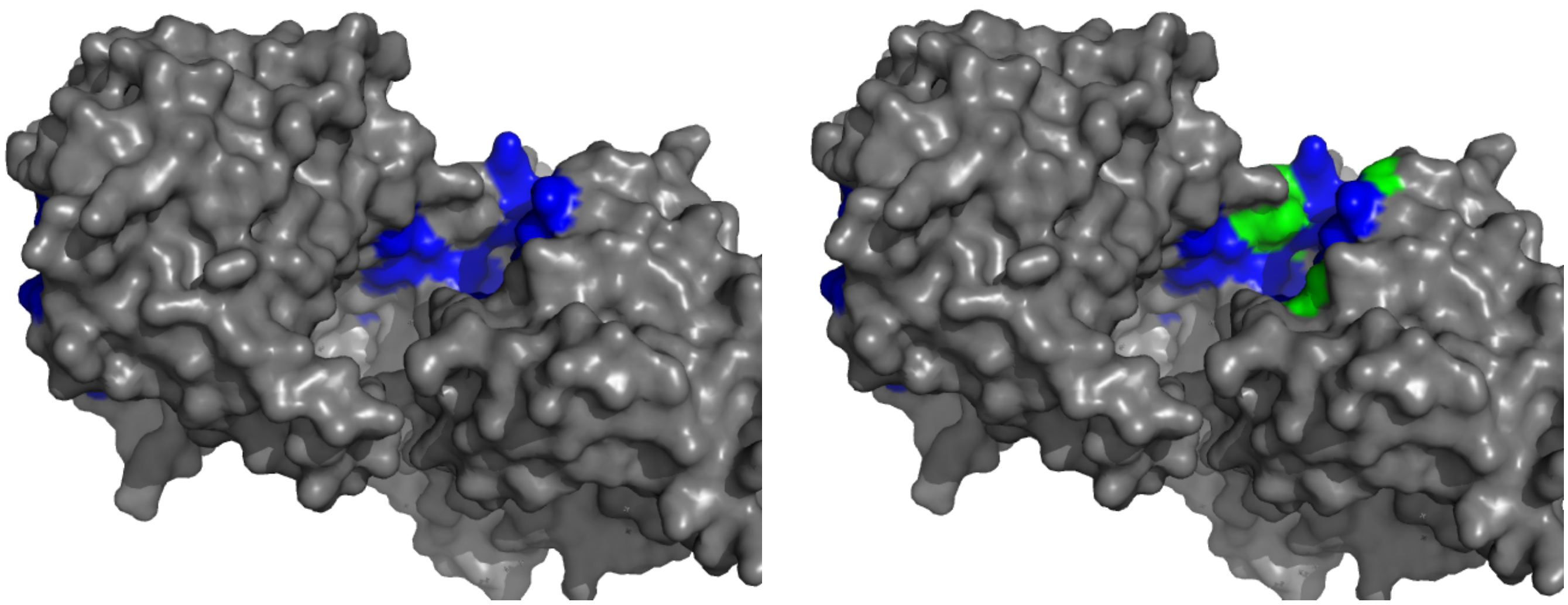

(a) Without smoothing

(b) With smoothing

Fig. S3. Example of residues added by smoothing classifier (GREEN: Gly25, Thr63, Phe65, Ala112, Met300, Phe304, Phe305) of a predicted pocket (BLUE). This is a 7o1iA structure from the CryptoBench test set (6).

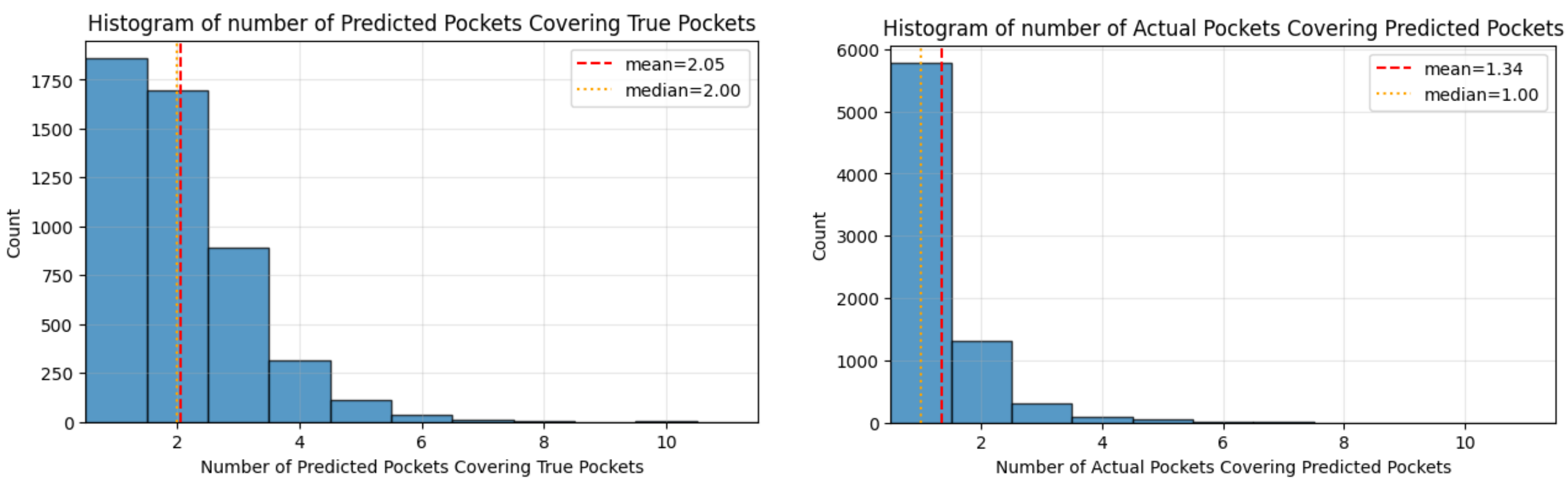

Fig. S4. Evaluation of predicted vs. actual pocket mapping of Seq2Pocket (GBS) on LIGYSIS. The **left diagram** shows the average number of predicted clusters per ground-truth pocket (fragmentation), while the **right diagram** shows the average number of ground-truth pockets per predicted cluster (merging). In an ideal one-to-one mapping, both means equal 1.0. While methods like DBSCAN reached high DCC recall, they showed excessive fragmentation (mean > 4). Our approach reduces this to 2.05.

Binding site type	Method	Train set	$DCC_{top\text{-}N}$	$DCC_{top\text{-}(N+2)}$	DCC_{MAX}
Small ($<$ 10 residues)	Seq2Pocket	$\text{sc-PDB}_{enhanced}$	**50.9%**	**58.9%**	**63.3%**
	Seq2Pocket	sc-PDB	41.7%	48.1%	51.1%
Large ($\geq$ 10 residues)	Seq2Pocket	$\text{sc-PDB}_{enhanced}$	**76.6%**	**81.8%**	**84.0%**
	Seq2Pocket	sc-PDB	70.7%	74.7%	76.5%

Table S1. Effect of data enhancement on small and large binding sites. To test the impact of training data enhancement, binding pockets in the LIGYSIS dataset were divided into two groups: *small* pockets containing fewer than 10 residues and *large* pockets containing 10 or more residues. DCC-based metrics were recomputed for each subgroup. Across all DCC variants, the improvements associated with training on $\text{sc-PDB}_{enhanced}$ are more pronounced for small binding sites.

Evaluation set	Method	Train set	$DCC_{top\text{-}N}$	$DCC_{top\text{-}(N+2)}$	DCC_{MAX}	% RRO
CryptoBench (CBS)	Seq2Pocket 650M	CryptoBench train set	69.5%	75.1%	75.1%	49.5%
	Seq2Pocket	*CryptoBench train set*	*70.4%*	*75.6%*	*76.5%*	*51.8%*
LIGYSIS (GBS)	Seq2Pocket 650M	sc-$PDB_{enhanced}$	61.0%	64.9%	65.8%	48.5%
	Seq2Pocket 650M, batch size=4	*sc-$PDB_{enhanced}$*	*52.0%*	*55.2%*	*55.9%*	*40.4%*
	Seq2Pocket	*sc-$PDB_{enhanced}$*	*62.0%*	*68.6%*	*71.9%*	*49.5%*

Table S2. Finetuning the ESM-2 (650M) model. To improve accessibility for settings without computational resources required for large models such as ESM-2 (3B), we repeated the finetuning procedure using smaller ESM-2 (650M) variant. These models exhibit a modest decrease in pocket-level performance compared to their 3B counterparts. We also retrained the 650M version with increased batch size on the GBS task, however it didn't lead to further performance improvement.

Evaluation set	Method	Scoring method	Train set	$DCC_{top\text{-}N}$	$DCC_{top\text{-}(N+2)}$	DCC_{MAX}	% RRO
COACH420	**Seq2Pocket**	**Sum of squares**	**sc-PDB$_{enhanced}$**	**89.8%**	**97.1%**	**98.1%**	**70.0%**
	Seq2Pocket	Mean	sc-PDB$_{enhanced}$	75.5%	93.1%	98.1%	70.0%

Table S3. Evaluating the Seq2Pocket (GBS) model on the COACH420 dataset. Table presents two methods for computation of the final pocket ranking: 1. Simple average of the prediction scores, 2. Sum of squares over the prediction scores. For datasets such as COACH420, which prioritises big (possibly orthosteric) binding sites (3), sum of squares is the superior ranking method, surpassing the original method by 14% in $DCC_{top\text{-}N}$. DCC_{MAX} and RRO are unchanged between the two ranking methods, as the ranking method only change the ordering of the results.

Evaluation set	Method	Train set	Filter	$DCC_{top\text{-}N}$	$DCC_{top\text{-}(N+2)}$	DCC_{MAX}	% RRO
LIGYSIS (GBS)	Seq2Pocket	sc-$PDB_{enhanced}$	Max. 40% identity	60.3%	65.7%	66.8%	47.9%
	Seq2Pocket	*sc-$PDB_{enhanced}$*	*Identical UniProt IDs removal*	*62.0%*	*68.6%*	*71.9%*	*49.5%*

Table S4. To see potential data leakage between the $scPDB_{enhanced}$ and LIGYSIS datasets, we further filtered the $scPDB_{enhanced}$ dataset down to max. 40% sequence indetity against the LIGYSIS datasets. The performance impact of this step was marginal - around 2% decrease in $DCC_{top\text{-}N}$ and less than 4% decrease in $DCC_{top\text{-}N+2}$. The filtering step has reduced the $scPDB_{enhanced}$ down to 9,927 unique structures.

Evaluation set	ProBiS z-score	DCC_{MAX}
LIGYSIS	no ProBiS hit	65.5%
	$z < 2$ (low similarity)	66.5%
	$z \geq 2$ (high similarity)	87.4%
	Full LIGYSIS	*71.9%*

Table S5. DCC MAX arranged by pocket structural similarity to the training set. For each LIGYSIS test pocket, we prescreened sc-PDB$_{\text{enhanced}}$ for its top-10 most similar structures to the test structure using a Foldseek (10), then used pairwise ProBiS (11) pocket alignment to compare the test pocket against every training pocket from those top-10 hits, keeping the best match. The resulting z-score defines a low-similarity ($z < 2$) and a high-similarity ($z \geq 2$) subset of LIGYSIS; "no ProBiS hit" is the subset of low-similarity pockets for which no candidate produced a significant local alignment at all. $DCC_{\text{MAX hit-rate}}$ is reported for each subset alongside the full LIGYSIS test set.

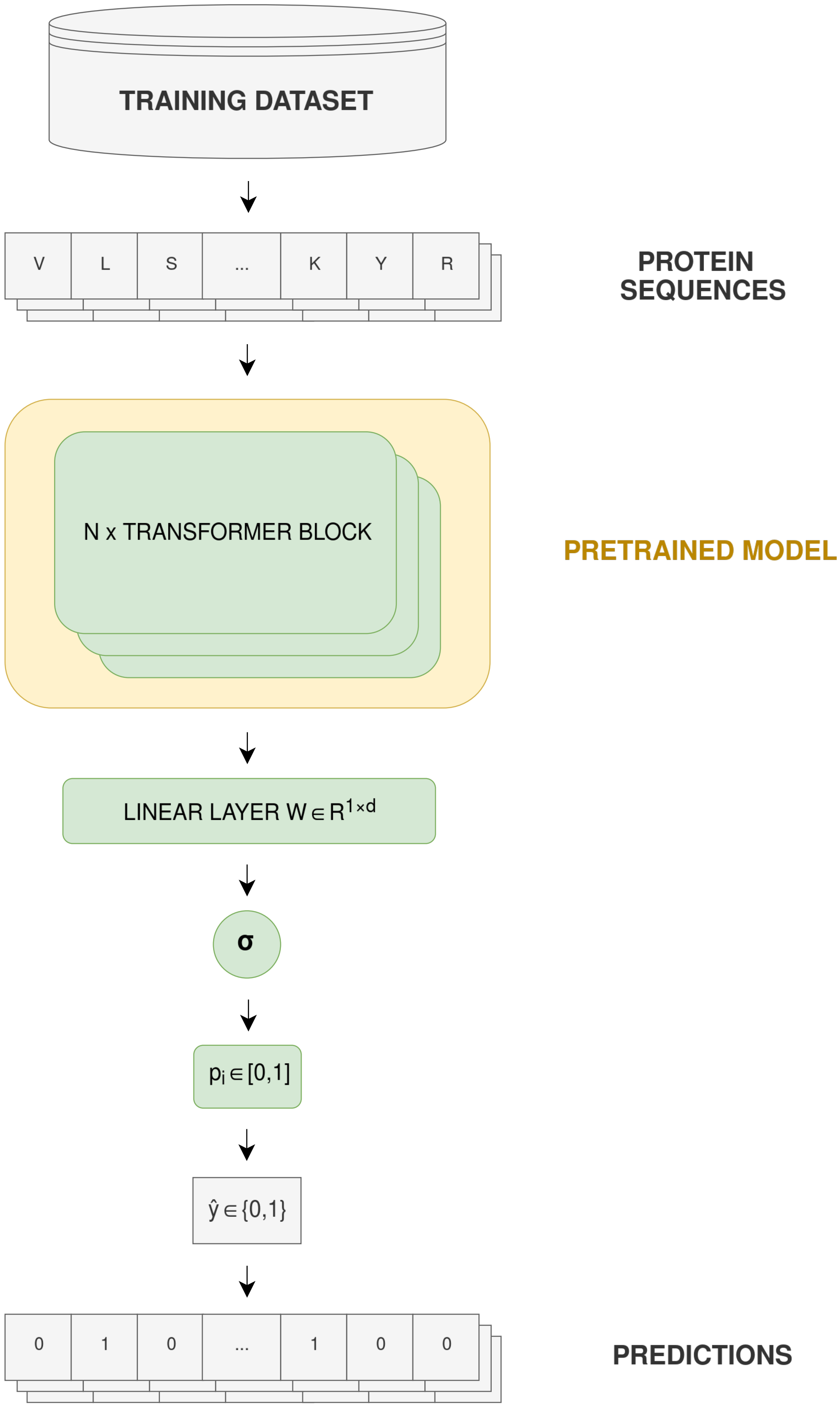

Fig. S5. Protein language model (pLM) finetuning scheme. During finetuning, the pLM (ESM2-3B) receives sequences from the training dataset - here either CryptoBench (6) for CBS prediction or sc-PDB (7, 8). for GBS prediction. Next, the original classification head is removed and replaced by a binary classification head for binding site prediction, see Linear Layer. Subsequently, the output of this layer is passed to a Sigmoid function (σ). This produces a probability $p_i \in [0, 1]$, which is binarized using a decision threshold into a single binary prediction for each residue in the sequence. Here, decision threshold was set to 0.7.

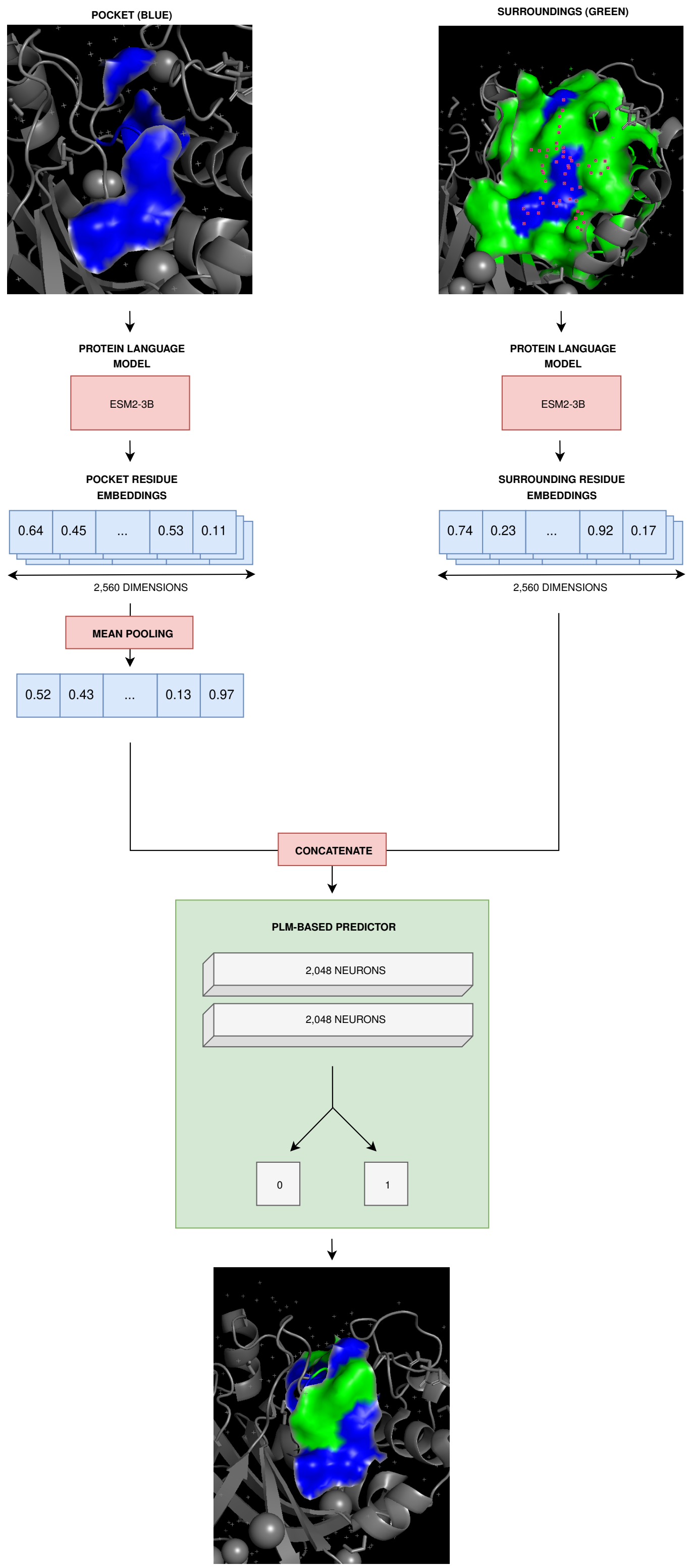

Fig. S6. Architecture of the smoothing classifier. The classifier processes two distinct inputs: (1) a global pocket representation, calculated as the mean average of all initial pocket residue embeddings (blue regions in the top-left and top-right picture), and (2) individual embeddings for all candidate residues located in the proximity of the predicted pocket (green region in the top-right picture). For each candidate, the model outputs a probability $p_i \in [0, 1]$ representing the likelihood of that residue being part of the pocket surface. These probabilities are binarized using a decision threshold of 0.4 to determine the final pocket membership. Final pocket is depicted on the bottom picture, where blue denotes the originally predicted residues, and green depicts the newly added residues.

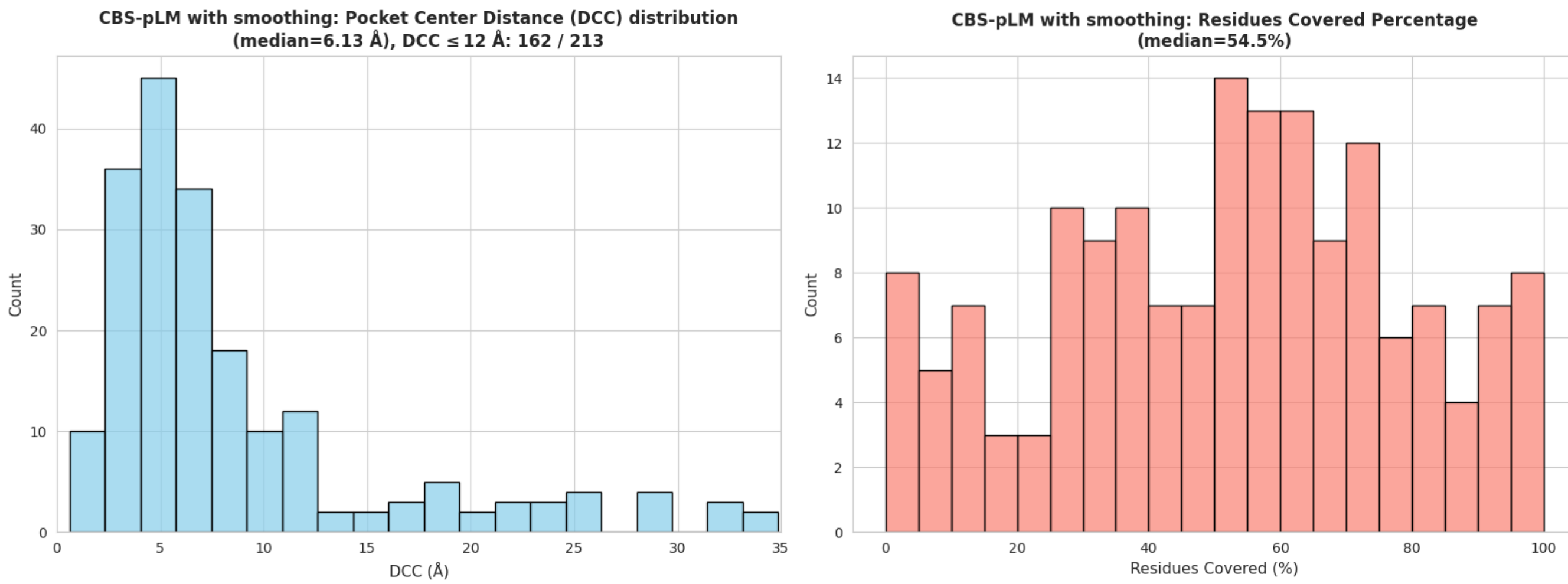

(a) Performance of CBS-finetuned pLM on the CryptoBench test set.

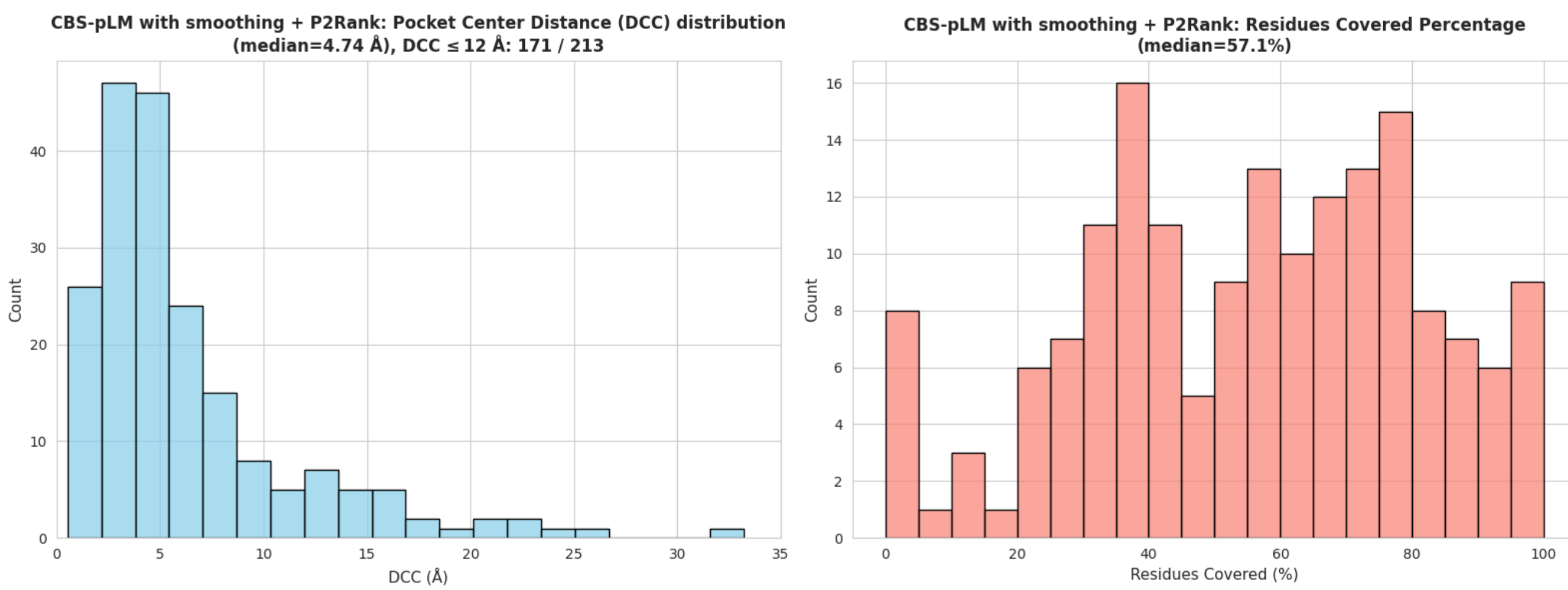

(b) Performance of integrated pLM and P2Rank (9) predictions on the CryptoBench test set.

Fig. S7. Performance impact of integrating P2Rank with CBS-finetuned pLM predictions. The left histogram displays the distribution of DCC_{MAX}, and the right histogram shows the distribution of RRO. Integration of P2Rank results with pLM predictions improves performance, resulting in a 4% increase in DCC_{MAX} and a 3% improvement in *median* RRO.

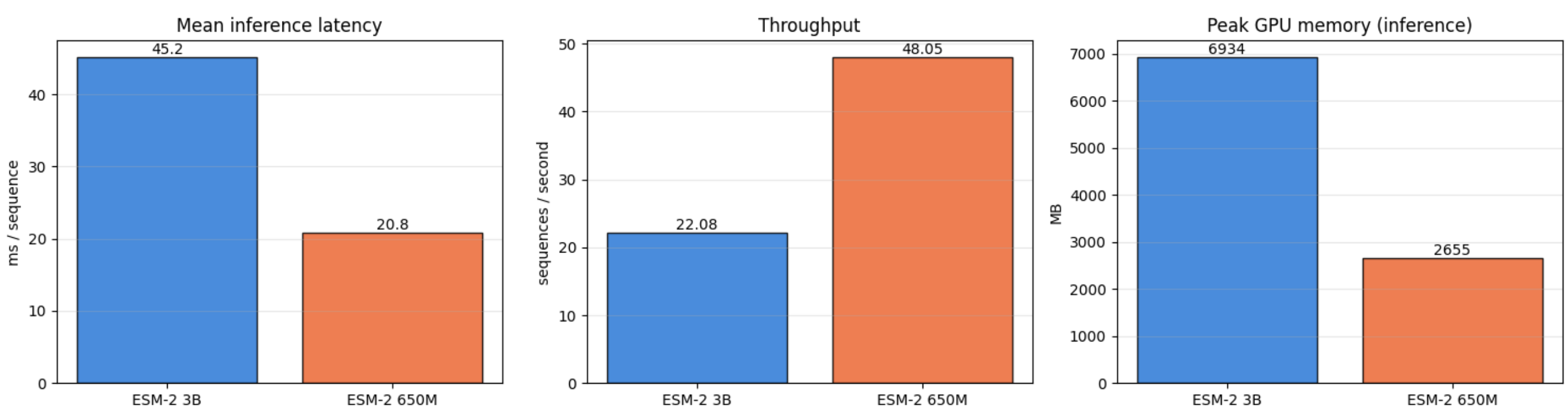

Fig. S8. Inference latency, throughput and peak GPU memory for the ESM-2 3B vs. 650M. The ESM-2 650M achieves $\tilde{2}$x lower inference latency and $\tilde{2}$x higher throughput than the 3B version on the NVIDIA L40 40GB GPU. The measurement was conducted on the first 100 LIGYSIS structures, with first 5 used only for warm-up.
